# Repeated Multiview Imaging for Estimating Seedling Tiller Counts of Wheat Genotypes Using Drones

**DOI:** 10.34133/2020/3729715

**Published:** 2020-09-07

**Authors:** Lukas Roth, Moritz Camenzind, Helge Aasen, Lukas Kronenberg, Christoph Barendregt, Karl-Heinz Camp, Achim Walter, Norbert Kirchgessner, Andreas Hund

**Affiliations:** ^1^ETH Zurich, Institute of Agricultural Sciences, Universitätstrasse 2, 8092 Zurich, Switzerland; ^2^Delley Samen und Pflanzen AG, Route de Portalban 40, 1567 Delley, Switzerland

## Abstract

Early generation breeding nurseries with thousands of genotypes in single-row plots are well suited to capitalize on high throughput phenotyping. Nevertheless, methods to monitor the intrinsically hard-to-phenotype early development of wheat are yet rare. We aimed to develop proxy measures for the rate of plant emergence, the number of tillers, and the beginning of stem elongation using drone-based imagery. We used RGB images (ground sampling distance of 3 mm pixel^−1^) acquired by repeated flights (≥ 2 flights per week) to quantify temporal changes of visible leaf area. To exploit the information contained in the multitude of viewing angles within the RGB images, we processed them to multiview ground cover images showing plant pixel fractions. Based on these images, we trained a support vector machine for the beginning of stem elongation (GS30). Using the GS30 as key point, we subsequently extracted plant and tiller counts using a watershed algorithm and growth modeling, respectively. Our results show that determination coefficients of predictions are moderate for plant count (*R*^2^ = 0.52), but strong for tiller count (*R*^2^ = 0.86) and GS30 (*R*^2^ = 0.77). Heritabilities are superior to manual measurements for plant count and tiller count, but inferior for GS30 measurements. Increasing the selection intensity due to throughput may overcome this limitation. Multiview image traits can replace hand measurements with high efficiency (85–223%). We therefore conclude that multiview images have a high potential to become a standard tool in plant phenomics.

## 1. Introduction

Research in field phenotyping has profited noticeably from recent advances in image processing which accelerated the implementation of sensor based crop monitoring. Nevertheless, field phenotyping is still regarded as the bottleneck for urgently needed crop genetic improvement [1]. Resilience to climate change has become a major issue in crop cultivation. In 2016 for example, 301 million tons of cereals were produced in Europe, 44.7% of which was wheat. Wet conditions and lack of solar irradiation in Northern Europe and hot and dry conditions in Southern Europe led to a loss of 13.5 million tons cereals compared to 2015 [2]. The influence of changing climatic conditions on the production of agricultural crops in Europe is ambiguous: in Northern Europe, the duration of the growing season is increasing due to higher temperatures and the flowering date of wheat has already advanced two to four days per decade since 1985 [3]. By contrast, heat and a reduction in precipitation are reducing the crop yield in Southern Europe [4]. Furthermore, yield variability between years is expected to increase due to the augmentation of extreme climatic events.

To ensure stable wheat production, one constantly needs to adapt the production systems to the changing climatic conditions, and breeders need to modify and improve wheat varieties permanently. Nevertheless, wheat yields are stagnating in Europe [5, 6]. To breed higher yielding wheat varieties, it is necessary to gain information on how and when the different yield components in a plant are formed [7]. While this need for reliable information on yield components is known since decades, the lack of high-throughput field phenotyping methods has hindered breeders to draw conclusions on genotype-environment interactions so far. Consequently, measuring final grain yield on large plots in late breeding stages is still regarded as the most effective phenotyping method. Nevertheless, it is crucial to phenotype earlier breeding stages in order to increase genetic gain [8]. In these early breeding stages, small plot sizes prevent robust yield measures and call for methods that examine single yield components.

Main yield components for wheat are the number of plants per square meter, the number of shoots with fertile ears per plant, and the number and weight of grains per ear [9]. The number of plants and ear-bearing shoots is related to plant emergence, tillering intensity, and tiller abortion, processes that are influenced not only by management decisions such as sowing density but also by environmental covariates [10]. The potential number of grains per ear—determined by the formation of the terminal spikelet—is linked to the time point when stem elongation starts [11], another process that is highly related to environmental parameters [12–15]. Evaluating these traits (beginning of stem elongation, plant, and tiller count) manually is very time-consuming, laborious, and prone to error.

High-throughput field phenotyping (HTFP) may represent a solution to the drawbacks of manual methods [1]. The use of color imaging (red, green, and blue (RGB)) in combination with unmanned aerial systems (UAS) or ground-based system—often called “phenomobiles” [16]—provides possible HTFP implementations at affordable costs. For wheat, successful attempts with RGB based methods were made to estimate plant counts under field conditions using problem-specific feature extraction (so-called “hand-crafted” traits) [17–19], machine learning [20], and deep learning [17]. Nevertheless, all of these methods require images with very detailed ground sampling distances (GSD < 0.5mm) at early growth stages, which limits scalability to large breeding experiments, as such methods cannot yet be applied with the GSDs achievable with drones.

For the estimation of tiller numbers, methods were developed that are based on spectral reflectance [21–23] and RGB indices [24–27]. For these methods, strong empirical correlations between tiller counts and vegetation indices were found. Nevertheless, none of the studies indicated the applicability of these methods in breeding. A notable exception is the work of Jin et al. [26]. The authors estimated ear-bearing shoot counts after harvest using images of stem residuals, thereby achieving heritabilities of up to 0.8, which indicates a high potential of such methods for plant breeding.

Remote sensing methods to estimate the growth stage (GS) beginning of stem elongation (BBCH 30, from now on referred to as GS30) [28] are rare: Kronenberg et al. [12, 13] used terrestrial laser scanning height measurements to determine 15% of final height as proxy for GS30 and reached a heritability of 0.85. Other published methods to remotely estimate the beginning of stem elongation are not known to us.

In UAS-based 2-D imaging, an area on the ground is captured from multiple angles. Depending on how the data is processed, different types of information can be extracted [29]. In a previous work, we developed a method to take advantage of image overlaps produced in UAS-based photography and used viewing geometry effects to estimate plant physiological traits [30]. Comparable approaches, but with the purpose to enrich training for deep learning, were simultaneously developed by Liu and Abd-Elrahman [31]. Liu et al. [32] demonstrated *in silico* that multiview observations of green fraction measured with RGB sensors may serve to derive important crop model parameter. In this study, we built on our previous work presented in Roth et al. [30].

**Figure 1:**
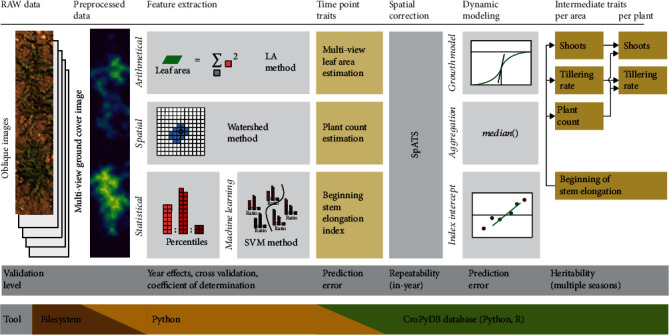
Phenotyping strategy including preprocessing, feature extraction, time point traits, spatial correction, dynamic modeling, and finally intermediate level traits. Graphs represent exemplary visualizations of the applied methods (LA: apparent leaf area; SVM: support vector machine).

The aim of this study was to predict plant count, tiller count, and the beginning of stem elongation using UAS-derived RGB images in a breeding context, which requires high-throughput and minimal user interaction. To accomplish this, we developed a new remote sensing product: *multiview ground cover images* (Figure 1). We then applied machine learning to predict GS30, and building up on this growth stage determination method used growth stage-sensitive parametric crop models to predict plant and tiller count.

The prediction of these traits was embedded in a HTFP strategy inspired by van Eeuwijk et al. [33]. Feature extraction from images was followed by automatized spatial correction of plot values using geospatial coordinates and subsequent modeling of the growth dynamics. We finally tested the quality of our methods as breeding tools by assessing repeatability, heritability, and relative efficiency.

## 2. Methods

### 2.1. Plant Material and Experimental Sites

Experiments were performed in two consecutive years (2018 and 2019) in a total of three year-site combinations, i.e., at two experimental sites: the field phenotyping platform site of ETH Zurich “FIP” [34] (Lindau Eschikon; Switzerland; 47.449 N, 8.682 E; 556 m a.s.l.) and the plant breeding site of Delley Samen und Pflanzen AG (Delley, Switzerland; 46.918 N, 6.979 E; 500 m a.s.l.). In the following, the year-site combinations are called “FIP18” and “FIP19” for the FIP site, and “Delley19” for the Delley site. The experiments were part of the regular testing of advanced breeding material of Agroscope (Nyon, Switzerland)/Delley Samen und Pflanzen AG (Delley, Switzerland) and consisted of 36 elite winter wheat genotypes per year-site. Because of the variety testing character, the set for 2018 differed from the set for 2019 by five genotypes.

Year-sites consisted of plots (experimental units in a row-range arrangement with spatial coordinates) enriched with block factors and genotypes. Here, we define range as the sowing direction, i.e., the sowing machine moves within rows across ranges. Details about the experimental designs, soil, and management can be found in Supplementary Materials C (available here).

Meteorological data was obtained from a weather station next to the experimental field (50 m) for the site FIP and from a public Agrometeo weather station (http://www.agrometeo.ch/, Agroscope, Nyon, Switzerland) in proximity (800 m) for the site Delley. Air temperature *T* was recorded 0.1 meter above the ground every ten minutes and averaged per hour to *T*_*h*_. From this data, growing degree days (GDDs) [35] were calculated in dependency of days after sowing (DAS) as

(1) $$\begin{array}{c} \text{GDD}\left(\text{DAS}\right)=\overset{\text{DAS}}{\underset{d=1}{\sum }}\overset{24}{\underset{h=1}{\sum }}\left\{\begin{array}{cc} \frac{T_{d,h}-T_{\text{base}}}{24}, & T_{d,h}>T_{\text{base}},\\ 0, & T_{d,h}\leq T_{\text{base}}, \end{array}\right. \end{array}$$

where *T*_base_ was assumed to be 0 °C [36].

In addition to GDD, the GDD difference relative to the beginning of stem elongation (ΔGDD_GS30_) was calculated as the difference between actual GDD and GDD needed to reach GS30 for each specific plot (GDD_GS30_):

(2) $$\begin{array}{c} \Delta \text{GD}{\text{D}}_{\text{GS}30}=\text{GDD}-\text{GD}{\text{D}}_{\text{GS}30}, \end{array}$$

where negative ΔGDD_GS30_ values indicate time points before and positive values time points after the beginning of stem elongation.

### 2.2. Manual Reference Measurements

Three categories of manual measurements were taken: measurements that were performed at all three year-sites (beginning of stem elongation), measurements taken at year-sites FIP18 and FIP19 (shoot count, plant count, and freezing damage), and measurements taken only at year-site FIP19 (phyllochron). Below, we give details on measurements following this sequence.

#### 2.2.1. All Year-Sites: Beginning of Stem Elongation

GS30 was determined by destructively measuring the distance between the basal node and the first extending node for three to five representative plants per time point and plot. When this distance reached ten millimeters, the plant was defined to be in the stem elongation stage [28, 37]. This ground truth data was collected three times between April 13 and April 19, 2018, for FIP18 for one replication; eight times between March 29 and April 22, 2019, for FIP19 for two replications; and 15 times between March 25 and April 10, 2019, for Delley19 for two replications. Since the beginning of stem elongation phase at FIP18 was very short and only three measurement dates could be performed, the measured distance between the first and the basal nodes was linearly interpolated to estimate the day when the stem elongation started. In contrast, for FIP19 and Delley19, estimated days correspond directly to measurement time points. As the measurement intervals for all measurements were between two and three days, the measurements presumably have the same uncertainty in days, which corresponds to approximately 20 to 30 GDDs.

#### 2.2.2. FIP18 and FIP19: Plant Count, Shoot Count, and Freezing Damage

**Figure 2:**
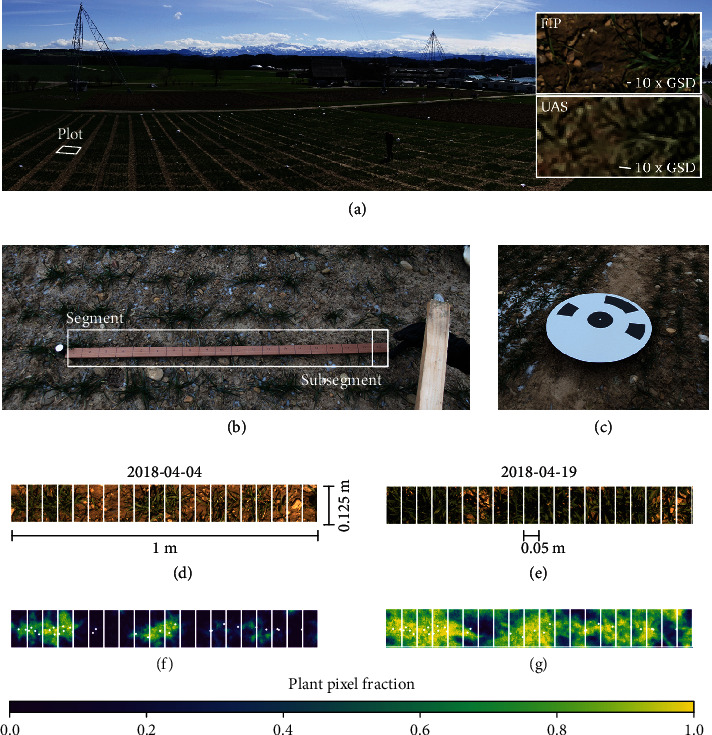
Field experiment at the FIP site viewed from the corner of Lot 3 with a visualization of resulting ground sampling distance (GSD) for images taken by the FIP and UAS (a), segment and subsegment labeling in field with micro ground control points with 20 mm diameter at borders of a 1 m row and yard stick with marked 0.05 m subsegment (b), coded ground control points with 0.5 m diameter (c), images taken by the FIP dolly from 3 m distance with a GSD of 0.6 mm (d, e), and equivalent multiview images based on images taken by the UAS from 28 m distance with a GSD of 3 mm (f, g) displaying plant pixel fractions. These fractions indicate the probability of pixels to show plant parts. Fractions close to one correspond to dense canopies or plant parts close to the ground; values close to zero correspond to soil parts; intermediate values correspond to erect or sparse plant parts.

To count plants and shoots and capture their spatial context, plots were subdivided in 20 subsegments each for one replication per year-site. A segment centered in the fourth sowing row of each plot with a length of 1 m was defined. The two ends of this segment were marked with micro ground control points (GCPs) by pegging wooden sticks into the soil (Figure 2(b)). Each segment was then further divided in subsegments with a size of 0.05 × 0.125m each using a displaceable yard stick (Figures 2(b), 2(d), and 2(e)).

Plant counts in subsegments were performed once per year-site at a stage where tillers were still distinguishable from main shoots. For FIP18, this was March 9, 2018, and for FIP19, February 13, 2019.

Shoots were counted manually in each subsegment and were defined as having emerged when the pseudostem was clearly visible. Shoot count measurements at the subsegment level of one replication were performed at ten time points for FIP18 and at six time points for FIP19; for FIP18 between March 14 and April 20, 2018; and for FIP19 between February 13 and April 23, 2019. In addition, to allow in-year repeatability calculations, shoot counts at segment level (1 m row) instead of subsegment level were performed for two replications of year-site FIP19 on March 28 and April 22, 2019.

Not all shoots initiated during the tillering phase may result in final ear-bearing shoots as tiller abortion is common in wheat [38]. In order to provide an estimate of abortion effects, ear-bearing shoots were counted manually per subsegment for year-site FIP18 shortly before harvest (July 9, 2018).

Due to very low temperatures between February 18 and March 3, 2018, some varieties suffered freezing damage, which was recorded on plot level on March 15, 2018, according to a score scale [39]. The scoring numbers ranged from 1 for excellent resistance to freezing to 9 for poor resistance to freezing.

To have an estimate of leaf area variations at early growth stages, measurements of leaf length from collar to tip (*L*) and maximum width (*W*) were performed on March 25, 2018, on each most expanded leaf per subsegment. Leaf area (LA) was then calculated using the approach proposed by Miralles and Slafer [40]: LA = *L* × *W* × 0.835.

#### 2.2.3. FIP19: Phyllochron and Plant Emergence

Phyllochron (defined as “the interval between similar developmental stages of leaves on the same culm”; [41]) and time point of plant emergence were determined using leaf stage measurements. The main stems of three plants per genotype were marked in early spring, and leaf stages following Haun [42] were determined weekly from February 18 to April 18, 2019. Linear regressions were used to extract genotype specific phyllochron and time point of emergence from leaf stage data.

### 2.3. Remote Sensing Campaigns

#### 2.3.1. UAS Campaigns

Fields at all three year-sites FIP18, FIP19, and Delley19 were prepared with crosswise GCP arrangements with 12 × 18m spacing, and GCP positions were measured using a GNSS differential global position system (R10, Trimble Ltd., Sunnyvale, U.S.A.) with swipos-GIS/GEO RTK (real time kinematic) correction (Federal Office of Topography Swisstopo, Wabern, Switzerland), resulting in a horizontal accuracy of 8 mm and a vertical accuracy of 15 mm. Coded round GCPs with a diameter of 0.5 m were used at the four corners of fields (Figure 2(c)), while at all other positions, uncoded squared GCPs with size 0.2 × 0.2m were placed. Both coded and uncoded GCPs consisted of UV prints on aluminum DIBOND plates (Mydisplays GmbH, Burscheid, Germany) placed 0.1 m above the ground using 0.55 m long soil anchor bolts (KSF E 89x550-E60 and KSF G 66x550-1xM8, Krinner GmbH, Walperswil, Switzerland).

For image acquisition, a mirrorless interchangeable-lens camera with a full frame sensor of 6000 × 4000 pixel (Sony *α*9 Model ILCE-9, Sony Corporation, Tokyo, Japan) equipped with a prime lens with a focal length of 55 mm and a maximum aperture of f/1,8 (Sonnar T∗ FE 55 mm F1,8 ZA, Sony Corporation, Tokyo, Japan) was mounted on a Matrice 600 Pro drone (SZ DJI Technology Co. Ltd., Shenzhen, China) using a Ronin-MX gimbal (SZ DJI Technology Co. Ltd., Shenzhen, China) to minimize off-nadir views and rotation blur effects. Flight planning was performed using PhenoFly Planning Tool [43], while DJI GS Pro (SZ DJI Technology Co. Ltd.) served as an autopilot for flight implementation. Camera and flight parameters were set as follows: shutter speed 1/16,000 s, ISO lower than 6,400, aperture between f/2.8 and f/7.1 (depending on illumination conditions), flight height 28 m, flight speed 1.8 m s^−1^, percent end lap 92%, and percent side lap 75%. These settings led to a GSD of 3 mm, restricted motion blur to ≤5%, reduced the signal-to-noise ratio to <27 dB [44], and ensured a GCP recover frequency of >70% for photos that showed one or more GCPs. According to PhenoFly Planning Tool [43], views of the plot centers had zenith angles in a range of 0-20° with the highest frequencies at 10°. Azimuth angles had the highest frequencies around 110° and 70° with local minima at 90° and a global minima at 0° (row direction); recovery rates were at 40 images per plot center and GCP (Supplementary Materials B, Figure 12).

For FIP18, 13 flights were performed between March 14 and May 1, 2018; for FIP19, 12 flights were performed between February 26 and April 18, 2019; and for Delley19, eight flights were performed between February 14 and April 11, 2019.

#### 2.3.2. FIP Campaigns

At the site FIP, the field phenotyping platform FIP [34] was operated on a regular basis. The system captured high-resolution RGB images with a GSD of 0.6 mm taken at 3 m distance to the ground with a EOS 5D Mark II sensor equipped with a 35 mm lens (Canon Inc., Tokyo, Japan). These images served as basis to manually identify the exact spatial position of single plants, a prerequisite to optimize watershed parameters for the plant count method (Section 2.5.2, Equations (10) and (11)).

As a preparation for manual plant identification in images, RGB images taken at seven consecutive dates in 2018 (March 5 to April 19, 2018) and three consecutive dates in 2019 (October 31, 2018; November 12, 2018; and February 22, 2019) were georeferenced using the position of micro GCPs marking the border of 1 m segment rows (see Section 2.2.2). A custom Python 3.6 script (e.g., [45]) was used to allow the user to identify and click positions of single plants in FIP images (source code available, see Section Additional Points, Point A1). After marking plant positions, captured image coordinates were translated to plot coordinates to simplify further processing with UAS-based multiview images (see the following section).

### 2.4. Multiview Image Generation

**Figure 3:**
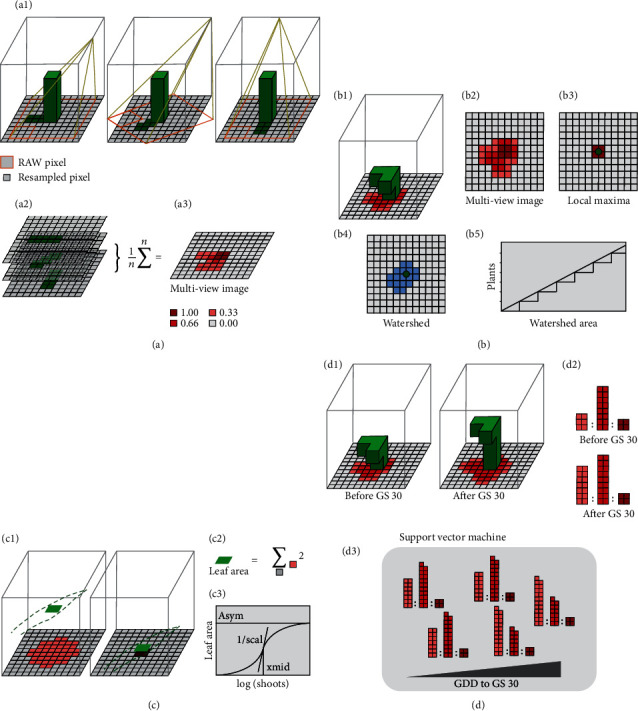
Schematic representation of multiview ground cover image preprocessing (a) and strategies for plant count (b), tiller count (c), and beginning of stem elongation (GS30, d) feature extraction methods.

All UAS campaigns were processed in a custom developed, uniform workflow that consisted of four major steps (Figure 3(a)): (1) camera exposure position and orientation determination; (2) identification of plots in individual images; (3) segmentation of individual images in pixel classified as plant or soil; and (4) aggregation of plot-based image cutouts to multiview images. In the following, each of these steps is described in detail (source code available, see Section Additional Points, Point 1–3).

#### 2.4.1. Camera Exposure Position and Orientation Determination

Individual oblique images from UAS flight campaigns were preprocessed with the structure-from-motion (SfM) software Agisoft PhotoScan Professional 1.4.2 and Agisoft Metashape 1.5.2 (Agisoft LLC, St. Petersburg, Russia) in order to derive exposure station position and orientation (Figure 3(a)). For the sparse point cloud processing, “keypoint limit” was set to 40,000 and “timepoint limit” to 4,000. Automatic GCP detection was used in an automated two-step process: first, coded GCPs were detected and matched with known coordinates using coded names. This step led to a roughly georeferenced point cloud. Second, uncoded GCPs were detected and matched with known coordinates of the closest uncoded GCPs. This second step led to a precisely georeferenced point cloud.

For the first flight campaign per year-site, the SfM process was continued with dense point cloud processing to get a digital surface model of the soil. The processing settings were “quality” to “high” and “depth filtering” to “mild.” Mesh and digital elevation model (DEM) were generated using a GSD of 3 mm, “surface type” was set to “height field”, interpolation was enabled, and a DEM and a RGB orthomosaic were exported as geoTiff. The DEM was further smoothed using a moving window median filter with window size 5 × 5. For all other campaigns, the SfM process was terminated after sparse point cloud processing, as DEM and orthomosaic were not needed for further processing.

#### 2.4.2. Identification of Plots in Individual Images

Once for each year-site, measured coordinates of the field corners were used to generate polygons that represent shapes of individual plots minus a buffer of 0.25 m. The buffer served two distinct purposes: (1) it should prevent sampling the wrong plot due to inaccurate positioning of plot polygons in images, and (2) it should prevent sampling image parts where plots overlap due to viewing geometry effects. The minimum buffer size *B* thereby corresponds to

(3) $$\begin{array}{c} B=\tan\left(\frac{\text{AOV}}{2}\right)h_{\text{canopy}}+E_{\text{ref}}, \end{array}$$

where AOV is the maximum angle of view of the imaging system, *h*_canopy_ the canopy height, and *E*_ref_ the georeferencing precision. Using a buffer size of 0.25 m therefore allows to monitor canopies with a maximum height of 0.41 m if using an imaging system with 40° AOV and assuming a georeferencing precision of 0.1 m [46], as done in this study. Subsequently, the RGB orthomosaic of the first campaign was used to manually adjust positions of plots and therefore integrate positioning shifts during sowing.

For all campaigns, after exposure station estimation, an approach similar to Roth et al. [30] was used to generate plot masks for each individual oblique image. In contrast to Roth et al. [30], we replaced the forward ray-tracing process with a custom Agisoft Metashape Python script that reverses the process. The script takes plot polygons in geojson format and a DEM as input, evaluates *Z* coordinates of plot corners using the DEM, and back projects XYZ world coordinates of plot polygon corners to images, thereby producing individual masks for each image. Image masks were saved as geojson files that contained plot identification numbers as attributes.

#### 2.4.3. Segmentation of Individual Images in Pixel Classified as Plant or Soil

For image segmentation in plant and soil pixel, we trained a random forest classifier using predictors based on 12 bit RGB values, four additional 12 bit color spaces, and two color indices. As wheat leaves in early stages are only a few millimeters wide, images with a GSD of 3 mm will contain major fractions of mixed pixels. The built-in RGB Bayer matrix of digital cameras will further intensify this effect, as only every second green value of pixel positions corresponds to a true measurement. Therefore, only pixel positions that correspond to green pixels in the Bayer matrix were taken for training and prediction, and red and blue values were interpolated for these pixel positions using the values of two corresponding adjacent pixels. Complementary color spaces (XYZ, sRGB, HSV, and La∗b∗) and plant-sensitive color indices (ExR and ExG; [30]) were derived as additional predictors for these green pixel positions.

The random forest classifier was implemented and trained with 380 samples from three different campaigns using the Python module scikit-learn [47]. The number of estimators was set to 55, minimum split size to 4, minimum forest leaf size to 6, maximum number of features per decision to 6, and maximum depth per decision tree to 95.

Our approach of using only green pixel positions for training and prediction resulted in missing values in the predicted image at blue and red pixel positions. These missing value positions were filled performing a morphological “opening” operation using a 3 × 3 pixel window, which additionally reduced noise in images. The resulting segmented binary images (segImg) were saved in the TIFF format.

#### 2.4.4. Aggregation of Plot-Based Image Cutouts to Multiview Images

As the next step, segmented images containing the same plot were collected and processed jointly. Thereby, pixels outside plot borders were masked first. Then, to facilitate further processing, pixels were resampled with threefold resolution (to reduce quality loss and artifacts caused by round-off errors) in a coordinate system with parallel axes to plot borders (plot coordinate system):

(4) $$\begin{array}{c} \text{segIm}{\text{g}}_{\text{res}}\left(x,y\right)=\text{segImg}\left(M\left[\begin{array}{c} x\\ y \end{array}\right]\right),x\in \left\{1,2,\cdots ,\frac{3\times \text{plo}{\text{t}}_{l}}{\text{GSD}}\right\},y\in \left\{1,2,\cdots ,\frac{3\times \text{plo}{\text{t}}_{w}}{\text{GSD}}\right\}, \end{array}$$

where plot_*l*_ and plot_*w*_ represent the length and width of the plot in millimeters, GSD the ground sampling distance, *M* the affine transformation matrix from a plot coordinate system to an image coordinate system, and segImg_res_ the resampled image. The change to a plot coordinate system thereby eliminated the overhead of geospatial allocation: while processing georeferenced images requires geographic information system- (GIS-) related software, processing images in plot coordinates can be done using any image processing software.

In a final step, all resampled images of each plot showing pixel classified as plant or soil were summed up pixel-wise and the result was divided by the number of images:

(5) $$\begin{array}{c} \text{mvImg}\left(x,y\right)=\frac{1}{n_{\text{Img}}}\overset{n_{\text{Img}}}{\underset{i=1}{\sum }}\text{segIm}{\text{g}}_{\text{res},i}\left(x,y\right), \end{array}$$

where *n*_Img_ is the number of oblique images (some of them close to nadir) for the corresponding plot. This process resulted in an image showing intensity values in the range of zero to one, the multiview ground cover image, further called multiview image mvImg (Figures 3 (a3) and 2(f)–2(g)).

### 2.5. Feature Extraction

In order to parameterize growth stage-dependent models for the prediction of plant and shoot counts based on physical appearance, the growth stage itself needs to be determined beforehand. Therefore, we first choose a machine learning approach based on statistical values extracted from multiview images to predict beginning of stem elongation (Figure 3(d)). Building up on this prediction, two individual models were built for the other traits: for plant count, we used a combination of spatial feature extraction and growth modeling (Figure 3(b)). In contrast, a combination of arithmetical calculation and growth modeling (Figure 3(c)) was used for shoot count. For implementation details on each approach and source code, please refer to the Section Additional Points (Point 4) below.

#### 2.5.1. Beginning of Stem Elongation

Feature extraction for the determination of the time point of GS30 was done using a machine learning approach based on visible ground cover in multiview images. The approach was based on the assumption that structural changes in the pseudostem erection phase will correlate with changes related to the beginning of stem elongation [48] (Figure 3 (d1)). These structural changes will lead to a change in the distribution of pixel fraction percentiles in multiview images (Figure 3 (d2)) following

(6) $$\begin{array}{c} \text{mvG}{\text{C}}_{i}=\frac{1}{n}\overset{n}{\underset{j=1}{\sum }}\left\{\begin{array}{c} 1,\text{mvImg}\left(j\right)>i,\\ 0, \end{array}\right. \end{array}$$

where mvGC_*i*_ is a pixel fraction percentile with the threshold *i*—now called multiview ground cover percentile, *j* represents a specific pixel on the multiview image, and *n* represents the total number of pixels. We therefore presumed that the change in multiview ground cover percentile distribution can be used to train a support vector machine regressor (Figure 3 (d3)).

To validate the approach and determine prediction errors, ten equally distributed multiview ground cover percentile values (Equation (6)) in the range of 10 to 100 were extracted for each plot and time point and complemented with ΔGDD_GS30_ based on manual GS30 measurements (Section 2.2.1). With these predictor and response pairs (filtered for a range of ±20ΔGDD_GS30_), a regressor support vector machine was trained in R [49] using the packages kernlab [50] and caret [51]. Based on a prior hyperparameter tuning, cost was set to 32 and gamma to 0.125. Tenfold cross-validations for genotype-based training/test splits and 3-fold cross-validations for year-site-based training/test splits followed training.

The custom developed phenotyping data processing infrastructure used in our research group (CroPyDB) is based on Python. To implement the developed approach as a high-throughput method and determine repeatability and heritability values, we trained a regressor support vector machine in Python using scikit-learn [47] with the same hyperparameter and training data set as for the validation and performed predictions for all year-sites. GS30 prediction error was defined as

(7) $$\begin{array}{c} E_{\text{GS}30}=\text{GS}30-\hat{\text{GS}30}, \end{array}$$

where GS30 corresponds to the manual reference measurement in GDD and $\hat{\text{GS}30}$ to the estimated value in GDD.

#### 2.5.2. Plant Count

To extract plant count numbers from multiview images, a spatial approach based on local maxima and watershed area sizes was used. The three underlying concepts of this approach are as follows:

1. It was assumed that a certain multiview ground cover percentile (mvGC_*i*_) correlates well with plant counts (*N*_plants_) (Figure 3 (b1) and (b2):

(8) $$\begin{array}{c} N_{\text{plants}}\approx a\times \text{mvG}{\text{C}}_{i}+b, \end{array}$$

where *a* and *b* are parameters of the linear regression to predict plant counts. Nevertheless, the exact intensity percentile is expected to be sensitive to physical appearance of plants and therefore year effects. Additionally, the performance of multiview ground cover percentiles as the predictor for plant count will depend on the growth stage of plants and therefore days to GS30.

(2) It was assumed that local maxima counts in multiview images correlate with true plant counts (Figure 3 (b1) and (b)):

(9) $$\begin{array}{c} N_{\text{plants}}\approx f_{\text{localmax}}\left(I_{\text{peak}},d_{\min},\text{mvImg}\right), \end{array}$$

where *f*_localmax_ extracts the number of local maxima with plant pixel fractions higher than *I*_peak_ and distances larger than *d*_min_ from each other. However, the approach was suspected to be sensitive to clumping of plants, where multiple adjacent plants may result in a single local maximum.

(3) To overcome the disadvantages of concept one and two, we combined both using watershed regions: if utilizing the best multiview ground cover percentile *i* as threshold to build watershed regions, the size of the region may correlate well with the sum of plants in that region in dependence of the growth stage (Figure 3 (b4) and (b5)):

(10) $$\begin{array}{c} \left\{A_{1},\cdots ,A_{n}\right\}=f_{\text{watershed}}\left(I_{\text{peak}},d_{\min},i,\text{mvImg}\right), \end{array}$$

(11) $$\begin{array}{c} {\hat{N}}_{\text{plants}}=\overset{n}{\underset{j}{\sum }}a_{W}\times A_{j}+b_{W}, \end{array}$$

where *A*_*n*_ are the areas of all watershed regions of mvImg that consist of plant pixels with factions larger than *i* around peaks with minimal plant pixel fractions *I*_peak_ and minimum distance *d*_min_. Parameters *a*_*W*_ and *b*_*W*_ are the slope and intercept of the linear regression, respectively, to predict plant counts based on watershed region sizes.

To get true plant counts for each watershed region, locations of plants manually identified in high-resolution FIP images (Section 2.3.2) were used to determine which set of plants corresponds to which watershed region. Consequently, we could fit a linear model (Equation (11)) to watershed region sizes and corresponding manual plant counts in these regions and evaluate the parameters *a*_*W*_ and *b*_*W*_ for five, ten, and 15 days before GS30.

A plant count prediction method based on Equations (10) and (11) and evaluated parameters *a*_*w*_ and *b*_*W*_ was implemented in Python using the package scikit-learn [47]. Using this method, plant count predictions were performed for all year-sites. Plant count prediction error *E*_plants_ was defined as

(12) $$\begin{array}{c} E_{\text{plants}}=N_{\text{plants}}-{\hat{N}}_{\text{plants}}, \end{array}$$

where *N*_plants_ corresponds to the manual reference measurement and ${\hat{N}}_{\text{plants}}$ to the estimated value.

#### 2.5.3. Shoot Count

Feature extraction for shoot counts was done using an arithmetic approach based on the apparent leaf area: depending on the distance between a leaf part and the ground, the footprint characteristics in multiview images will differ (Figure 3 (c1)). Nevertheless, summing up pixel intensities of multiview images will correspond to an estimation of leaf area (Figure 3 (c2)). The apparent leaf area LA per plot was estimated from multiview images as

(13) $$\begin{array}{c} \text{LA}=\overset{n}{\underset{j=1}{\sum }}\text{mvImg}{\left(j\right)}^{2}, \end{array}$$

where *j* represents a specific pixel on the multiview image and *n* the total number of pixels. Image values were squared before summing up to compensate for a skewed distribution of zenith angles towards off-nadir views, which is typically found in UAS-based images [30, 43].

After leaf area calculation, shoot counts were derived from LA based on physiological constrains of tiller and leaf development [52] (Figure 3 (c3)):

1. The number of leaves on main stems (*N*_*l*,*MS*_) depends on the phyllochron (*P*) and on GDD at emergence (*t*_0_):

(14) $$\begin{array}{c} N_{l,MS}=P\times \left(\text{GDD}-t_{0}\right)=f_{1}\left(\text{GDD}\right). \end{array}$$

In this study, *P* and *t*_0_ are regarded to have no significant differences in the elite genotype set (see e.g., Duan et al. [25]; Ochagavía et al. [53] and Section 3.1). *N*_*l*,*MS*_ can therefore be expressed as a function of GDD only (*f*_1_).

(2) The number of leaves on the *n*th tiller (*N*_*l*,*Ti*_*n*__) depends on the tiller number (*n*_*Ti*_) and number of leaves on the main stem:

(15) $$\begin{array}{c} N_{l,Ti_{n}}=a\times N_{l,MS}-b\times n_{Ti}=f_{2}\left(\text{GDD},n_{Ti}\right), \end{array}$$

where Abichou et al. [52] consider *a* and *b* as genotype-independent constants, by what *N*_*l*,*Ti*_*n*__ can be expressed as a function of GDD and *n*_*Ti*_ only (*f*_2_).

**Figure 4:**
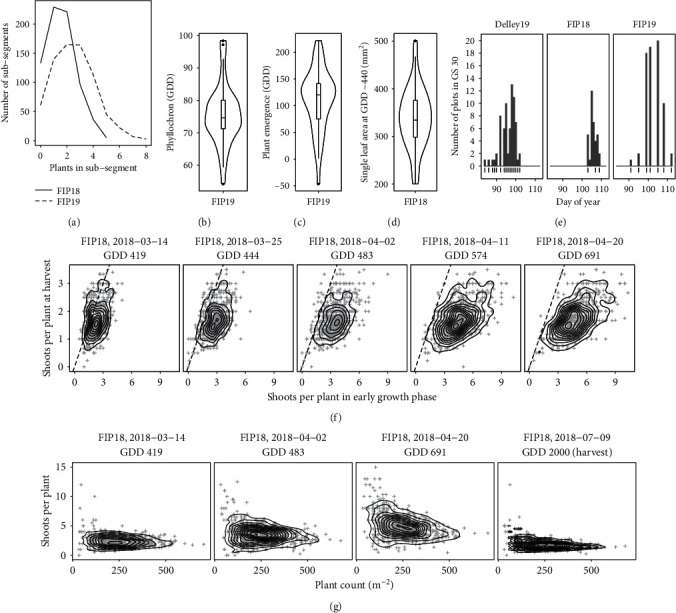
Manual reference measurements of plant counts (a), phyllochron (b), plant emergence time point (c), leaf area of single leaves around GDD 440 (d), beginning of stem elongation (GS30) (e), contour plot of shoots per plant at specific growing degree days (GDDs) around stem elongation and at harvest (f), and contour plot of shoots per plant for different measured plant densities, specific GDDs, and at harvest (g). The boxes in (b, c, d) show the 25 and 75% percentiles, the solid line represents the median, the whiskers show the 5 and 95% percentile, and the violins show the kernel density distribution. Contours in (f, g) are density contours, dashed lines—1 : 1 lines.

(3) If assuming that leaves in early growth stages before the beginning of stem elongation have all the same area *A*_*l*_ (see Section 3, Figure 4(d)), the leaf area of a canopy (leaves on main shoots and tillers) can be expressed in the dependency of the number of main stems *N*_*MS*_, number of tillers *N*_*Ti*_, number of leaves for the *n*th tiller *N*_*l*,*Ti*_*n*__, and leaf area *A*_*l*_ as

(16) $$\begin{array}{c} \text{LA}=N_{MS}\times A_{l}\times \left(N_{l,MS}+\overset{N_{Ti}}{\underset{n=1}{\sum }}N_{l,Ti_{n}}\right). \end{array}$$

When inserting Equations (14) and (15) into (16),

(17) $$\begin{array}{c} LA=N_{MS}\times A_{l}\times \left(f_{1}\left(\text{GDD}\right)+\overset{N_{Ti}}{\underset{n=1}{\sum }}f_{2}\left(\text{GDD},n_{Ti}\right)\right), \end{array}$$

it becomes obvious that LA can be expressed as a function of the number of shoots *N*_*S*_ (as sum of *N*_*MS*_ and *N*_*Ti*_) and GDD:

(18) $$\begin{array}{c} \text{LA}=f\left(N_{MS},N_{Ti},\text{GDD}\right)=f\left(N_{S},\text{GDD}\right). \end{array}$$

Based on a visual inspection of manual reference measurement data, we approximated the relationship between LA, number of shoots (*N*_*S*_), and growth stage (GDD) by a logistic function:

(19) $$\begin{array}{c} \text{LA}=\frac{\text{Asym}}{1+\exp\left(\left(\text{xmid}-\mathrm{log1p}\left(N_{S}\right)\right)/\text{scal}\right)}, \end{array}$$

where log1p returns the natural logarithm of one plus the input. The asymptote Asym can be fixed to the LA value that corresponds to the maximal canopy cover value. We assumed a maximum canopy cover of 90% [54] for all genotypes based on the observation that values above 50% are very unlikely before GS30 and genotype specific variations in the asymptote therefore neglectable (Supplementary Materials B, Figure 9). To meet the requirements of Equation (18), the other two parameters xmid and scal therefore need to be in dependence to the growth stage. If assuming linearity, xmid and scal may be expressed in dependence to ΔGDD_GS30_ as

(20) $$\begin{array}{c} \text{xmid}=a_{\text{xmid}}\times \Delta \text{GD}{\text{D}}_{\text{GS}30}+b_{\text{xmid}}, \end{array}$$

(21) $$\begin{array}{c} \text{scal}=a_{\text{scal}}\times \Delta \text{GD}{\text{D}}_{\text{GS}30}+b_{\text{scal}}, \end{array}$$

where *a*_xmid_, *b*_xmid_, *a*_scal_, and *b*_scal_ are slopes and intercepts of the linear regressions. We estimated these parameters using a nonlinear fit of Equation (19) to ΔGDD_GS30_ groups with an aggregation window of ±12.5 GDD.

A shoot count prediction method based on Equations (19), (20), and (21) and evaluated values for *a*_xmid_, *b*_xmid_, *a*_scal_, and *b*_scal_ was implemented in Python, and shoot count predictions were performed for all year-sites. Shoot count prediction error *E*_shoots_ was defined as

(22) $$\begin{array}{c} E_{\text{shoots}}=N_{\text{S}}-{\hat{N}}_{\text{S}}, \end{array}$$

where *N*_S_ corresponds to the manual reference measurement and ${\hat{N}}_{\text{S}}$ to the estimated value.

### 2.6. Spatial Correction and Repeatability Calculation of Time Point Traits

After preprocessing of data (Section 2.4) and feature extraction (Section 2.5), calculated time point traits were spatially corrected. This correction yielded repeatability values and corrected plot-based trait values (Figure 1). For the spatial correction, spatial modeling of the R package SpATS [55] was applied to all time points and measurements that included at least two replications. The model thereby corresponds to

(23) $$\begin{array}{c} Y=\text{PSANOVA}\left(x,y\right)\;|\;R_{x}+R_{y}+G, \end{array}$$

following the syntax proposed by Piepho et al. [56]. PSANOVA is a smoothed bivariate surface defined over *x* and *y* coordinates in a metric coordinate system (EPSG:3395, WGS 84/World Mercator), *R*_*x*_ is the row effect, *R*_*y*_ is the range effect, *G* is the genotype effect, and “|” separates fixed from random effects. For the bivariate surface, the SpATS parameter number of segments (determining the number of internal knots of the surface) was chosen according to the number of plots in each direction, row, and range, multiplied with 2/3.

Correction was done for all plots in all year-sites (4 × 36 plots for FIP19 and Delley19, 2 × 353 plots for FIP18). Corrected plot-based values were calculated as the sum of best linear unbiased estimations (BLUEs) and residuals. Repeatability was determined per time point using generalized heritability [57] with the package SpATS.

### 2.7. Dynamic Modeling and Repeatability Calculation of Intermediate Level Traits

Corrected time point traits were further processed after spatial correction to yield intermediate level traits. The term “intermediate level trait” in this research refers to plot and year-site-specific parameters that summarize nondestructive repeated measurements on the same observational unit, i.e., a plot in our case. This concept refers to the one proposed by van Eeuwijk et al. [33] with the exception that the intermediate level traits were derived on a plot level rather than on a genotype level.

Different methods for dynamic modeling were chosen for the three different traits: for plant counts, the median of three spatial corrected time point measurements was taken. For shoot counts, an inverse exponential model was fitted to plot based time point data:

(24) $$\begin{array}{c} N_{S,t}=N_{S}-\exp\left(-a\times \Delta \text{GD}{\text{D}}_{\text{GS}30}\right), \end{array}$$

where *N*_*S*,*t*_ corresponds to the number of shoots at a specific time point, *N*_*S*_ to the final shoot count before cessation (tiller abortion), and *a* to the tillering rate (Supplementary Materials B, Figure 10). Since training data for the development of the shoot count method were in a range of −200 ≤ ΔGDD_GS30_ < 0, values outside this range were filtered out before fitting Equation (24) to the data in order to prevent extrapolation. For year-site Delley19, not enough time points were left after filtering. Therefore, *N*_*S*_ was calculated as the median of tiller counts *N*_*S*,*t*_ and estimations for *a* skipped.

After fitting Equation (24), the extracted parameters final shoot count *N*_*S*_ and tillering rate *a* were further processed with plant count estimations to estimate final shoot count per plant and tillering rate per plant instead of area (Figure 1). Prediction errors for *N*_*S*_ and *a* were calculated by additionally fitting Equation (24) to the manual reference measurement data in order to extract reference values for *a* and *N*_*S*_.

To determine the exact timing of beginning of stem elongation, a linear regression was fitted per plot to estimated values in the range ΔGDD_GS30_ ± 20:

(25) $$\begin{array}{c} \hat{\Delta \text{GD}{\text{D}}_{\text{GS}30}}=a\times \text{GDD}+b. \end{array}$$

Values from campaigns having a repeatability lower than 0.5 were filtered out before fitting Equation (25) to the data. GS30 was then determined by evaluating the intersection of the right-hand side of Equation (25) with zero, GS30 = −*b*/*a* (Supplementary Materials B, Figure 11).

For all methods, prediction errors were calculated for rows of one meter, and Pearson's correlations were calculated on the same level. Repeatability was evaluated using the R package lme4 [58] to fit a linear mixed model and based on generalized heritability. The model used was

(26) $$\begin{array}{c} Y=R\;|\;G, \end{array}$$

where *R* is the replication effect, *G* is the genotype effect, and “|” separates fixed from random effects.

### 2.8. Heritability Calculation

After intermediate level trait extraction per year-site, generalized heritabilities over multiple year-sites were calculated using the R package lme4 [58] to fit a linear mixed model [57]. Different models were used for two cases:

1. Multiple replications, multiple years: *Y* = *Yr*/*R* | *G*.
2. One replication, multiple years: *Y* = *Yr* | *G*

In the two cases, *Yr* is the year effect, *R* is the replication effect, *G* is the genotype effect, “|” separates fixed from random effects, and “/” indicates nesting of factors. Generalized heritability was calculated for plots of the common set of 36 genotypes. Consequently, 2 × (353 − 36) plots showing noncommon genotypes at year-site FIP18 were filtered out before heritability calculation. In addition to the calculation for four replications, HTFP data sets were reduced to two and one replication, to make the resulting heritability values comparable to manual reference measurements.

### 2.9. Relative Efficiency Calculation

**Figure 5:**
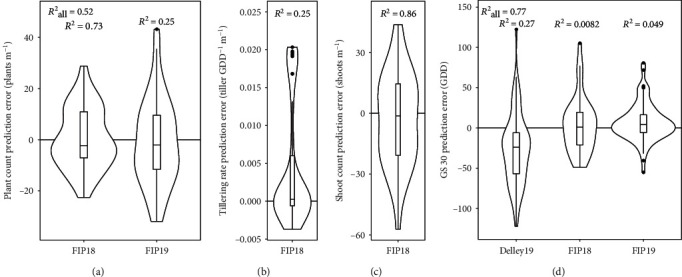
Prediction errors and determination coefficients (*R*^2^) for plant count estimations (a), shoot dynamics modeling (b, c), and beginning of stem elongation (GS30) (d). The boxes show the 25 and 75% percentiles, the solid line represents the median, the whiskers show the 5 and 95% percentile, and the violins show the kernel density distribution; *r* is Pearson's correlation.

Using HTFP methods to replace manual measurements in breeding programs follows the concept of indirect selection [59, 60]. The efficiency of indirect selection can be evaluated using relative efficiency (RE) estimations:

(27) $$\begin{array}{c} \text{RE}=\frac{h_{\text{HTFP}}\cdot r_{G}}{h_{\text{manual}}}, \end{array}$$

where *h*_HTFP_ is the square root of the heritability of the HTFP method, *h*_manual_ the square root of the heritability of the corresponding manual reference method, and *r*_*G*_ the genotypic correlation between the corresponding methods [59]. To estimate *r*_*G*_, we used the plot-based, phenotypic correlations for all available year-sites *r*_all_ (see Section 3, Figure 5).

## 3. Results

### 3.1. Insights Based on Manual Reference Measurements

Plant count measurements at the site FIP performed in 2018 and 2019 revealed that the homogeneity of plant emergence varied between the years (Figure 4(a))—emergence in 2018 was more homogeneous with few subsegments containing more than two plants, while in 2019, higher plant counts per subsegment were frequent. Leaf stage measurements in 2019 at the site FIP showed that phyllochron variation in the examined genotype set was small: values ranged from 54 to 98 GDD, while 50% of all values were in a narrow band between 70 and 80 GDD (Figure 4(b)). The time point of plant emergence based on leaf stage determination showed similar narrow ranges with 50% of all values between 75 and 140 GDD (Figure 4(c)). Leaf areas of most expanded single leaves ranged from 200 to 500 mm^2^ with 50% of all values between 300 and 380 mm^2^ (Figure 4(d)). Ranges for beginning of stem elongation time points varied largely between sites and years: while at year-sites Delley19 and FIP19 the beginning of stem elongation spanned a period of 20 days, at year-site FIP18, all genotypes reached stem elongation within a period of less than ten days (Figure 4(e)).

Final ear-bearing shoots per plant at harvest at FIP18 were—based on the visual assessment of Figure 4(f)—most closely related to shoot counts at the beginning of tillering, representing almost 1 : 1 the final number of spikes (~GDD 400; March 10, 2018; GS 21-24). During the tillering phase, considerably more tillers were produced for almost all plots, visually indicating an overproduction of shoots towards the beginning of stem elongation (~GDD 700; April 20, 2018; GS30). The number of shoots per plant covaried to some extent with plant counts towards beginning of stem elongation, but not at earlier phases or at harvest (Figure 4(g)).

### 3.2. Feature Extraction Model Parameterization

For plant counts, further details on the preliminary parameter estimation steps using approaches one and two (ground cover percentile and local maxima) can be found in Supplementary Materials E. The consequent combined watershed approach confirmed that the year effect was, in contrast to approaches one and two, not significant (Supplementary Materials B, Figure 8d; Supplementary Materials A, Table 3).

For the shoot count method, parameters scal and xmid were extracted using nonlinear fits to ΔGDD_GS30_ groups. ANOVA tests for scal and xmid confirmed that the year effect was not significant but the dependency on ΔGDD_GS30_ was essential (Table 1). Plotting xmid and scal versus ΔGDD_GS30_ revealed that assuming linearity (Equations (20) and (21)) was justified (Supplementary Materials B, Figure 9c-d).

### 3.3. Accuracy of Intermediate Level Traits in Comparison with Manual Measurements

After feature extraction, time point traits were further processed to intermediate level traits. In the following, we present prediction errors, RMSEs, relative RMSEs (rRMSEs), determination coefficients (*R*^2^), and cross-validations for all year-sites that included reference measurements for the specific traits.

For the plant count method, prediction errors were well centered around zero (Figure 5(a)). The RMSE for FIP18 was 11.8 plants m^−1^ (rRMSE = 23.3%) and for FIP19 17 plants m^−1^ (rRMSE = 33.7%). The inferior performance for FIP19 was also reflected in a moderate *R*^2^ (0.25) in comparison to a strong *R*^2^ for FIP18 (0.73), while the overall *R*^2^ was reasonably strong (0.52). Overall RMSE was 14.8 plants m^−1^ (rRMSE = 29.2%).

The dynamic modeling of shoot counts supplied two parameters: the tillering rate (*a*) and final shoot count (*N*_*S*_) (Figures 5(b) and 5(c), Equation (24)). Errors for tillering rates were small for 50% of all rows, but a group of outliers formed at an error of 0.02 tiller GDD^−1^ m^−1^. Plotting predicted values against reference measurements revealed that outliers had predicted rates close to zero (Supplementary Materials B, Figure 10b) which indicates a lack of tillering dynamic in the measurement period. RMSE was at 0.0089 tiller GDD^−1^ m^−1^ (rRMSE = 42.9%). The *R*^2^ was moderate (0.25).

Prediction errors for final shoot counts before cessation were well centered around zero with a RMSE of 23 shoots m^−1^ (rRMSE = 17.0%). The *R*^2^ was strong (0.86).

Characteristics of beginning of stem elongation prediction errors showed well-centered prediction errors for the year-sites FIP18 and FIP19 and a slight underestimation for year-site Delley19 (Figure 5(d)). RMSEs were at 51 GDD for Delley19, 35 GDD for FIP18, and 24 GDD for FIP19. The overall RMSE was at 39 GDD. Although ranges of prediction errors and RMSEs for the different year-sites were comparable, the *R*^2^revealed severe mismatches for FIP18 (0.008), but a moderate linear association for Delley19 (0.27) and a reasonably strong association for FIP19 (0.49). Tenfold cross-validations for genotype based training/test splits and threefold cross validations for year-site based training/test splits revealed RMSEs in the range of 52 to 90 GDD and rRMSEs between 10.6 and 18.5% (Table 2).

### 3.4. Repeatability of Time Point Traits in Comparison with Manual Measurements

Preprocessing, multiview segmentation, and feature extraction generated plot-based values for 34 measurement time points at three year-sites. In the following, we present repeatability values of HTFP methods for all time points and compare them—if available—with repeatability values of manual reference measurements.

**Figure 6:**
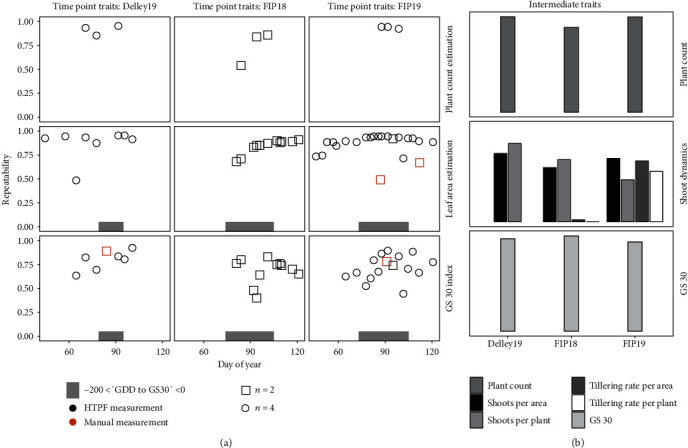
Repeatability values for time point traits before dynamic modeling (a) and year-site repeatability values for intermediate level traits based on time point traits after dynamic modeling (b). Intermediate level traits were calculated using different dynamics functions (see Section 2.7): for plant count, the median of time point plant count estimations, for shoot dynamics an inverse exponential model with parameters final shoots and tillering rate based on estimated shoot numbers using time point leaf area estimations, and for growth stage 30 (GS30) the intersection of a linear regression of time point GS30 index values with zero.

Repeatabilities for the multiview image based plant count method were high for all three year-sites and three time points per year-site and ranged between 0.84 and 0.96, except for the first campaign for FIP18 with a repeatability of 0.54 (Figure 6(a)). Subsequent analyses revealed operational problems for the corresponding underlying flight: the camera trigger sequence was interrupted for some flight lines, which partially led to lower overlaps. Nevertheless, we decided to keep the flight campaign in our data set to demonstrate the advantage of dynamic modeling based on repeated measurements.

For the leaf area estimation method that served as a proxy for shoot counts, repeatability was increasing over time for FIP18 and FIP19 and reached values of up to 0.96, while the values for Delley19 stayed at a constant high level between 0.88 and 0.96, except one low value at 0.49. After filtering for the corresponding range of -200 to 0 ΔGDD_GS30_, remaining values used for the dynamic modeling ranged between 0.88 and 0.96. Repeatabilities for manual shoot counting in 2019 at the site FIP were at 0.49 for the first measurement during the tillering phase and 0.67 for a second measurement after beginning of stem elongation. Hence, repeatabilities for manual reference measurements with two replications were lower than for the corresponding HTFP measurements with four replications. Nevertheless, HTFP leaf area estimation was only a proxy for shoot counts and comparability is therefore limited.

Repeatabilities for the multiview image based beginning of stem elongation method varied over time and year-sites: for Delley19, high values between 0.81 and 0.93 were calculated for later growth stages, while values for early measurements remained lower between 0.64 and 0.83. For FIP18, repeatabilities were all in the same range of 0.64 to 0.83 except for two lower values below 0.5 at early growth stages. For FIP19, values for early growth stages were lower than those for later stages but generally between 0.63 and 0.83, except for two low repeatability values below 0.5. Repeatabilities for manual GS30 measurements with two replications were at 0.89 for Delley19 and 0.78 for FIP19 and hence comparable to HTFP measurements with four replications.

### 3.5. Repeatability of Intermediate Level Traits in Comparison with Manual Measurements

Dynamic modeling resulted in plot-based values (see Section 2.7). In the following, we present repeatability results based on these plot-based values.

Repeatabilities for HTFP plant counts after dynamic modeling were high for all three year-sites (0.87 to 0.98) (Figure 6(b)). Repeatability of HTFP shoots per area estimations for the different year-sites ranged between 0.55 and 0.7. HTFP estimations were thereby comparable to manual shoot count measurements performed after beginning of stem elongation: HTFP repeatability for FIP19 (0.65) corresponds well to the one of manual measurements (0.67). When combining shoot estimations with plant counts to yield shoots per plant estimations, repeatabilities for Delley19 (0.8) and FIP18 (0.63) increased, but dropped for FIP19 (0.43).

Tillering rate values were only available for FIP18 and FIP19, while for Delley19, not enough time point measurements were available. All repeatabilities for FIP18 were close to zero, but repeatability for tillering rate per area for FIP19 was at 0.62 and tillering rate per plant at 0.51.

For the beginning of stem elongation, repeatability values for all year-sites were high (0.94 for Delley19, 0.97 for FIP18, and 0.92 for FIP19) and comparable to manual reference measurements (0.89 for Delley19, 0.78 for FIP19).

### 3.6. Heritability and Relative Efficiency of High-Throughput Traits

#### 3.6.1. Heritability in Comparison with Manual Measurements with the Same Number of Replications

**Figure 7:**
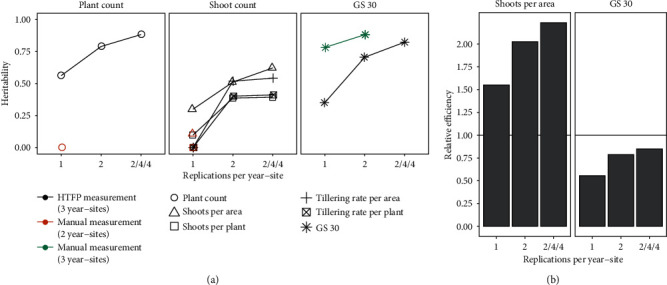
Multiple season heritability values of intermediate level traits (a) and relative efficiency of HTFP methods in comparison to manual measurements for intermediate level traits where the heritability of manual measurements was nonzero (b). For manual measurements, all year-sites had one (plant count, shoot count) and two (growth stage 30 (GS30)) replications. For high-throughput field phenotyping (HTFP) methods, values are given for the full number of replications (two for FIP18, four for FIP19, and four for Delley19, indicated as “2/4/4”) and for two reduced number of replications (“1” and “2”).

Based on plot-based intermediate level trait values of all three year-sites, heritability was calculated for HTFP methods as well as manual reference measurements. To allow direct comparison with manual reference measurements, replication numbers for HTFP methods were reduced to two and one replication for multiyear-site heritability calculation (Figure 7(a)).

For the plant count estimation method, the heritability based on one replication per year-site was 0.56, while the heritability for the manual reference measurements was zero. Heritability for the shoots per area estimation method with one replication was 0.3 and thus higher compared to the manual method (0.11), and the same applied for the shoots per plant method (0.1 versus zero). For tillering rate with one replication, HTFP and reference methods showed zero heritabilities. For the beginning of stem elongation estimation trait with two replications, the heritability of manual reference measurements (0.88) was superior to the one of the HTFP method (0.71).

#### 3.6.2. Increased Heritability due to Throughput

Throughput allowed increasing replication and year-site numbers for the HTFP methods. This advantage had clear effects on heritability (Figure 7(a)). For plant counts, shoots per area, and beginning of stem elongation, heritability increased continuously with increasing number of replications. The beginning of stem elongation thereby reached a heritability of 0.82, plant counts 0.88, and shoots per area 0.62. For tillering rates and shoots per plant, increasing replication numbers from two to four did not show any further improvements of heritability—values reached 0.39 for shoots per plant, 0.54 for tillering rate per area, and 0.41 for tillering rate per plant.

#### 3.6.3. Relative Efficiency

Relative efficiencies were calculated based on heritabilities for manual measurements (Section 3.6.1), heritabilities of HTFP methods (Section 3.6.2), and correlations between the methods (Section 3.3). As heritabilities of manual measurements for plant count, shoot per plant, and tillering rate where zero or close to zero, relative efficiency values for the corresponding HTFP methods were infinite. The relative efficiency for shoots per area started at 1.55 for one replication per year-site and increased to 2.23 for four replications per year-site. The relative efficiency for beginning of stem elongation increased from 0.55 to 0.85 with increasing number of replications.

## 4. Discussion

In this work, we presented multiview images as a novel UAS-based approach for high-throughput phenotyping. By exploiting the additional information contained in the multitude of viewing angles within the UAS images capturing an area on the ground, this approach enables the quantification of intrinsically hard-to-phenotype traits such as the beginning of stem elongation or tiller count [16]. A direct comparison between the multiview image approach and standard UAS approaches is difficult. In comparison to segmented single-view images (e.g. [19]), multiview ground cover images show intensity values (plant pixel fractions) rather than just binary values (plant versus soil). This characteristic allows applying methods such as spatial algorithms, which do not work with single-view images. Consequently, a direct comparison of the performance of single-view images with multiview images on the level of extracted traits is only possible if comparable methods exist for both products. Nevertheless, all existing plant count methods known to us rely on GSDs smaller than 0.5 mm [16], which is hardly achievable with currently available drone systems. In addition, neither a shoot count method nor a GS30 method based on images is known to us. Therefore, to give evidence of the key characteristic of multiview images, we validated our methods with manual measurements with high temporal and spatial resolution.

All following feature extraction steps were designed to work with multiview images. Dense point processing—a calculation intensive task typically done in SfM processing to yield 3-D information—was not required for time point trait extraction. Processing costs of less than half a day per flight campaign and minimal manual interaction requirements (see Supplementary Materials D) indicated a high usability of multiview images in applications that require high throughput. The required ground sampling distance of 3 mm thereby allowed capturing images efficiently, a substantial advantage over other ground-based methods with comparable precision.

The multiview image approach saves the time for generating the dense point cloud. Nevertheless, canopy heights extracted from a point cloud could represent important additional information, e.g., to train a machine learning predictor. The decision not to generate dense point clouds therefore represents a trade-off between saving processing time and not taking advantage of the full potential of data. If processing time is not limiting, combining multiview images with canopy heights may be of advantage.

Requirements on drone flights for multiview images are more rigid than for other remote sensing products. Images should have equally distributed zenith angles in relation to plot centers, frequent views perpendicular to the row direction, and plots should appear at equal frequencies in images. Using proper flight planning is therefore essential.

For the quality of the multiview images, it is essential to apply an appropriate buffer size to plot polygons that considers important parameters such as maximum canopy height, angle of view, and georeferencing precision. These restrictions were taken into account by choosing a large buffer size of 0.25 m. Furthermore, the precision of coregistration between multiple views will have an impact on the quality of multiview images. Our approach is based on the internal camera alignment of Agisoft Metashape, which it reports to be accurate in the range of one to two pixels. Consequently, the resulting image masks have a corresponding precision.

In a breeding context, a HTFP method is only of use if extracted traits allow reliable selection of targeted ideotypes. Hereafter, we therefore discuss the robustness of individual traits based on multiview images, followed by a discussion of implications for breeding.

### 4.1. Beginning of Stem Elongation

The beginning of stem elongation served as an important anchor point for the subsequent prediction of plant and shoot counts. The overall RMSE of 39 GDD indicates suitability to predict the beginning of stem elongation for year-sites and genotype sets where time points span multiple weeks. Nevertheless, if transitions happen in less than ten days, as in FIP18 between April 12 and April 20, 2018 (~100 GDD), this accuracy may be too low, even if the measuring frequency is increased considerably. This finding is supported by the low *R*^2^ at year-site FIP18. Surprisingly, repeatability values of intermediate level traits indicated a good useability for all year-sites including FIP18. Ultimately, higher multiple season heritability values for manual measurements than for the HTFP traits, as well as increasing RMSEs in cross-validation for unseen genotype/year-site sets, revealed the root of the problem: The used machine learning method showed a tendency to overfit and most probably also included other training characteristics that were not related to the target trait, e.g., differences in plant emergence homogeneity between the years at site ETH (Section 3.1, Figure 4(a)), differences in soil composition between the sites ETH and Delley (Supplementary Materials C), and differences in soil moisture between individual measurement time points. In a given year-site, this results in higher repeatability values, but over year-sites, heritability values fall short below the one of reference measurements. This lack of generalization may be reduced by using more training samples, and it clearly indicates advantages of hand-crafted traits over machine learning approaches if using small training data sets.

Nevertheless, the relative efficiency of up to 0.85 indicates a high potential of the method to distinguish early and late beginning of stem elongation in large-scale breeding experiments. To our knowledge, other studies reporting accuracy of HTFP GS30 estimates are not available. However, GS30 can also be estimated based on the dynamics of stem elongation using terrestrial laser scanning [12, 13]. The authors found considerable genetic variation for the beginning of stem elongation (defined as the GDD at which 15% of final height was reached) with a heritability of 0.82 across three growing seasons, which perfectly fits the heritability for four replications found in this study (0.82) but is superior to the heritability for two replications (0.71). Nevertheless, substantial differences exist between the study materials: While Kronenberg et al. [12, 13] examined a diverse set of approximately 330 common European winter wheat genotypes from a diverse range of breeding origin; we examined a set of 36 Swiss elite genotypes originating mainly from one single plant breeder. We therefore expect the heritability of the method developed by Kronenberg et al. [12, 13] to be lower for a genotype set that is more related to a breeding context.

Adapting the method of Kronenberg et al. [12, 13] to drone images by extracting the dynamics of height development with sufficient precision would be feasible (e.g. [46]) but would have two essential consequences on measurement and processing practice. First, it would require processing RGB images to dense point clouds, extracting plant heights from the difference between measured heights of a specific campaign and a soil height reference, and determining the time point where 15% of the final height is reached in a semiautomated way. Second, it would require continuing the height measurements throughout the whole growing season, thereby capturing the final height of plants, a prerequisite for the relative height calculation. While the first consequence would increase the calculation effort and reduce the throughput, the second consequence would effectively prevent breeders from drawing first conclusions on genotype performance in-season.

Beginning of stem elongation determination on multiview images on the other hand allows selecting genotypes in-season with high efficiency. Thereby, our results indicated that two replications per year-site are sufficient—increasing the replication number from two to four only slightly improved efficiency.

### 4.2. Plant Count

HTFP yielded good estimates of plant counts across year-sites with high repeatability and moderate prediction errors. However, the comparison of found prediction errors with other studies is difficult. Most other authors were choosing experimental designs that included sowing density and nitrogen treatments but only a few genotypes, which reduces the informative value of results for breeding. Under these restrictions, plant count prediction errors based on multiview images were comparable with the method of Liu et al. [18] who reported errors of 22% for experiments with similar sowing densities and five genotypes, while in this study we found prediction errors of 11.8 and 17.0 plants m^−1^ which corresponds to 23.3 and 33.7% (Figure 5). Liu et al. [19] reported superior performance with errors of 12.5% for three different genotypes and nitrogen treatments and Liu et al. [17] errors of 12% for few genotypes in sowing density treatments. It remains unclear to us whether applying the mentioned methods to breeding experiments would preserve these high precisions.

Jin et al. [20] on the other hand tested their method on breeding experiments but relied on a very fine ground sampling distance: Required flight heights for their method ranged between three and seven meters, and several flights were necessary to cover areas comparable with those of this study. Consequently, they reached accurate prediction errors of 14.31%. Breeding-related repeatability or heritability values were not provided. Heritability of plant counts may be affected by nongenetic factors, such as seed storage conditions. It is therefore expected that the heritability within a season is high (particularly, when there is a large variability in seed quality) but low across seasons.

In this study, heritability values for the HTFP method were considerably higher than those for manual reference measurements (Figure 7(a)). Differences in sampling size may have contributed to this finding: While we hand-sampled a one-meter row within the nine-rowed plot, which is representing not even 10% of the overall plot size, the HTFP method was based on the whole plot. For manual measurements, Jin et al. [26] proposed to use sample sizes of at least six meters if counting fixed lengths or to count 90 plants in total, which should then lead to errors <10%. Following this recommendation may have led to higher heritability values for manual measurement based genotype means, but the effort needed to perform such manual measurements would have been high. This finding points out the need for and benefit of HTFP methods. Furthermore, it gives implications for using small plot sizes in early breeding stages. Even if using phenomobiles taking images at early growth stages (e.g. GS 11-12) from proximity with a GSD of less than 0.1 mm as discussed by Hund et al. [16] and demonstrated by Liu et al. [18], severe inhomogeneity in small plot sizes may still prevent high heritability values. On the other hand, if using larger plot sizes, higher precision is most probably superfluous, as the variation due to inhomogeneity between rows of the same plot may be higher than the precision gain when using proximity measurements.

Irrespective of the proximity of the method, nonexisting heritabilities of manual measurements indicate a clear advantage of HTFP for breeding; manual measurements as done in this study are not suitable for breeding (Figure 7(a)). High heritabilities of plant count estimations based on multiview images on the other hand may empower breeders to use the method for multiple purposes, e.g., to calculate deviated traits such as tillers per plant in a HTFP manner as demonstrated in this study; to exclude seed lots with poor emergence performance from evaluations; or to screen for emergence behavior based on genotype-environment interactions or seed treatments.

### 4.3. Shoot Count

Available literature on shoot counts is limited—most studies included sowing density treatments based on few genotypes, only. When considered in a breeding context, the prediction efficiency of our method is most probably superior to other studies. While Phillips et al. [22] found an *R*^2^ of 0.74 for 310 measurements at 22 year-sites, which is comparable to our method (*R*^2^ = 0.86), Scotford and Miller [23] found a RMSE of ±125 shoots m^−2^, which is comparable with the prediction error of 16.9% of our method. Again, no breeding-related repeatability or heritability values were provided.

In our study, repeatabilities of shoot count estimation showed little variation over year-sites (Figure 6(b)). Repeatabilities of tillering rates on the other side indicated strong year effects. The high overall heritability of shoots per area and lower heritability of shoots per plant additionally indicate that a genotype-specific “desired” shoot density exists and significantly differs between genotypes. Compensation effects for sowing densities and emergence rates are well-known for wheat [10].

Heritability values for manual reference measurements were low to nonexisting, which led to high relative efficiencies for HTFP methods (Figure 7(b)). This finding indicates that using HTFP is of high advantage for breeders who aim to select ideotypes based on tiller dynamics. While two replications per year-site may provide a reasonable base for selection, higher replication numbers may not justify the additional effort.

The presented shoot count method was based on the assumption that genotypes in early growth stages have similar leaf areas. This simplification allowed developing a generalizable method with a strong *R*^2^ if compared to manual reference measurements. Nevertheless, there is evidence in the literature that the variation in leaf width is genotype specific (e.g., [61]). Incorporating leaf area as genotype-specific parameter in a future work may therefore further improve tiller count predictions.

### 4.4. Implications for Breeding

Multiview images used in this study allowed processing of UAS-based data in a high-throughput manner with minimal human interaction and in reasonable processing time. The required flight height of approximately 30 m above ground thereby enables breeders to screen larger breeding populations with precisions comparable to phenomobile approaches. However, flight time and battery capacity is still the limiting factor for experiments with large plot sizes: To fly an experimental field site with two replications of 350 genotypes and 1 × 1.5 m plot sizes took approximately 15 minutes in this study, which is at the absolute limit of battery capacity.

Nevertheless, as even individual rows can be monitored with the presented methods, HTFP may be applied in early-generation breeding gardens. Thus, on the same area, more than 3000 individual lines could potentially be screened, if each row was planted with a different variety. This would allow to increase the selection intensity by means of a larger number of genotypes being screened. As Rebetzke et al. [8] stated, HTFP should play a key role in allowing breeders to increase sizes of their breeding nurseries to finally increase genetic gain. We are not aware that tillering capacity or the beginning of stem elongation is routinely assessed in early generation-breeding programs yet. Thus, we consider the developed method also as a new selection tool in the breeder's toolbox.

Finally, combining the presented plant and shoot count methods with high-throughput ear count methods could supply ample information on mechanisms and genetic backgrounds of tiller formation and abortion. Having insights in such processes will contribute to the stabilization of grain yield even in rapidly changing climatic conditions.

## 5. Conclusion

High-throughput is a term at risk to become worn-out in crop phenotyping: While many studies propagate to present such high-throughput methods, few bring it to the stage where applicability can be assessed. Despite the increasing interest in crop phenotyping driven by the genomics community, throughput is still an issue, both on the level of data processing and on the level of data capturing.

In this study, we presented multiview images in combination with dynamic modeling of repeated measures as a new remote sensing product that may become key to combine throughput and precision for drone-based crop phenotyping. Since the method enriches the information content of images by integrating multiple views in one single plot-based product, it required moderate ground sampling distances (~3 mm), thereby allowing to screen large fields by means of drones. We used the turning point of the beginning of stem elongation as an anchor to parameterize developmental crop models, thus taking into account different developmental stages among genotypes. Using the example of plant count and shoot count combined with beginning of stem elongation, we demonstrated applicability of the approach to extract new target traits for breeding. We therefore conclude that multiview image processing in combination with developmental modeling may become a standard tool in crop phenotyping to meet the demands of throughput and precision.

## Figures and Tables

**Table 1:** ANOVA of linear models for parameters scal and xmid of the logistic leaf area versus tiller count model. Given are degrees of freedom (Df), sum of squares (Sum Sq), mean of squares (Mean Sq), *F* statistics (*F* value), and significance (Pr(>*F*)).

	Df	Sum Sq	Mean Sq	*F* value	Pr(>*F*)
Parameter: scal					
ΔGDD_GS30_	1	0.62	0.62	35.61	0.0001
Year	1	0.01	0.01	0.70	0.4198
ΔGDD_GS30_ : Year	1	0.01	0.01	0.68	0.4241
Residuals	12	0.21	0.02		

Parameter: xmid					
ΔGDD_GS30_	1	1.19	1.19	63.24	0.0000
Year	1	0.01	0.01	0.33	0.5775
ΔGDD_GS30_ : Year	1	0.01	0.01	0.68	0.4266
Residuals	12	0.23	0.02		

**Table 2:** Cross-validation (CV) results for training of beginning of stem elongation support vector machine. Results are root mean squared errors (RMSEs) in growing degree days (GDDs) and relative root mean squared errors (rRMSEs).

Training	Test	Test type	RMSE	rRMSE
All data	All data	10-fold CV	52.6	10.9%
Genotype subset	Unseen genotypes	10-fold CV	51.7	10.6%
Year-site subset	Unseen year-sites	3-fold CV	85.3	17.7%
Genotype subset in FIP18/FIP19 subset	Unseen genotypes in Delley19	10-fold CV	89.8	18.5%
Genotype subset in FIP18/Delley19 subset	Unseen genotypes in FIP19	10-fold CV	80.8	18.4%
Genotype subset in FIP19/Delley19 subset	Unseen genotypes in FIP18	10-fold CV	79.7	16.7%

## Additional Points

*Source Code*. Maintained source code for processing is publicly available on the ETH Zurich GitLab server (https://gitlab.ethz.ch/crop_phenotyping/PhenoFly_data_processing_tools); archived versions can be found in Roth [62, 63]. The GitLab repository includes following preprocessing steps: (1) Image mask generation (Standalone Agisoft Metashape Script) (https://gitlab.ethz.ch/crop_phenotyping/PhenoFly_data_processing_tools/ImageProjectionAgisoft). (2) Image segmentation with random forest (https://gitlab.ethz.ch/crop_phenotyping/PhenoFly_data_processing_tools/ActiveLearningSegmentation). (3) Multiview image generation (https://gitlab.ethz.ch/crop_phenotyping/PhenoFly_data_processing_tools/MultiViewImage). The GitLab repository furthermore includes the following trait extraction method: (4) Early growth trait extraction (https://gitlab.ethz.ch/crop_phenotyping/PhenoFly_data_processing_tools/EarlyGrowthTraitExtraction). Addition useful tools can be found here: (A1) Utils (https://gitlab.ethz.ch/crop_phenotyping/PhenoFly_data_processing_tools/Util). *Data*. Secondary data (plot-based values, BLUEs, BLUPs, repeatability, and heritability) are available at the research collection of ETH Zurich (https://www.research-collection.ethz.ch/) (Roth, [64]). Primary data of this research (RAW images) are available upon request; a sample data set is publicly available (Roth, [65]).

## Supplementary Materials

Supplementary Materials Supplementary Materials A: additional tables. B: additional figures. C: site description. D: high-throughput processing details. E: plant count method details.
