# Supplementary material for: Repeated Multiview Imaging for Estimating Seedling Tiller Counts of Wheat Genotypes Using Drones
Source: Plant Phenomics. 2020 Sep 7;2020:3729715. doi: 10.34133/2020/3729715 (PMC7706335; doi:10.34133/2020/3729715)
Supplement: Supplementary Materials — A: additional tables. B: additional figures. C: site description. D: high-throughput processing details. E: plant count method details. [file 3729715.f1.zip › 3729715.f1/S_A_Tables.pdf]

Supplementary Materials

A: Additional tables

Table 3: ANOVA of linear Model 1 (plant count as a function of 50th ground cover percentile, growth stage and year) and Model 2 (plant count as function of watershed area, growth stage and year). Given are degrees of freedom (Df), sum of squares (Sum Sq), mean of squares (Mean Sq), F statistics (F value) and significance (Pr(>F)).

Model 1	Df	Sum Sq	Mean Sq	F value	Pr(>F)
50th ground cover percentile	1	7763.42	7763.42	1750.68	0.0000
50th ground cover percentile : growth stage	2	5094.57	2547.28	574.42	0.0000
50th ground cover percentile : year	1	868.82	868.82	195.92	0.0000
Residuals	3721	16500.83	4.43		
Model 2					
Watershed area	1	5875.54	5875.54	2264.25	0.0000
Watershed area : growth stage	2	3469.76	1734.88	668.57	0.0000
Watershed area : year	1	0.62	0.62	0.24	0.6264
Residuals	4635	12027.43	2.59		
