# Supplementary material for: Repeated Multiview Imaging for Estimating Seedling Tiller Counts of Wheat Genotypes Using Drones
Source: Plant Phenomics. 2020 Sep 7;2020:3729715. doi: 10.34133/2020/3729715 (PMC7706335; doi:10.34133/2020/3729715)
Supplement: Supplementary Materials — A: additional tables. B: additional figures. C: site description. D: high-throughput processing details. E: plant count method details. [file 3729715.f1.zip › 3729715.f1/S_B_Figures.pdf]

Supplementary Materials

B: Additional figures

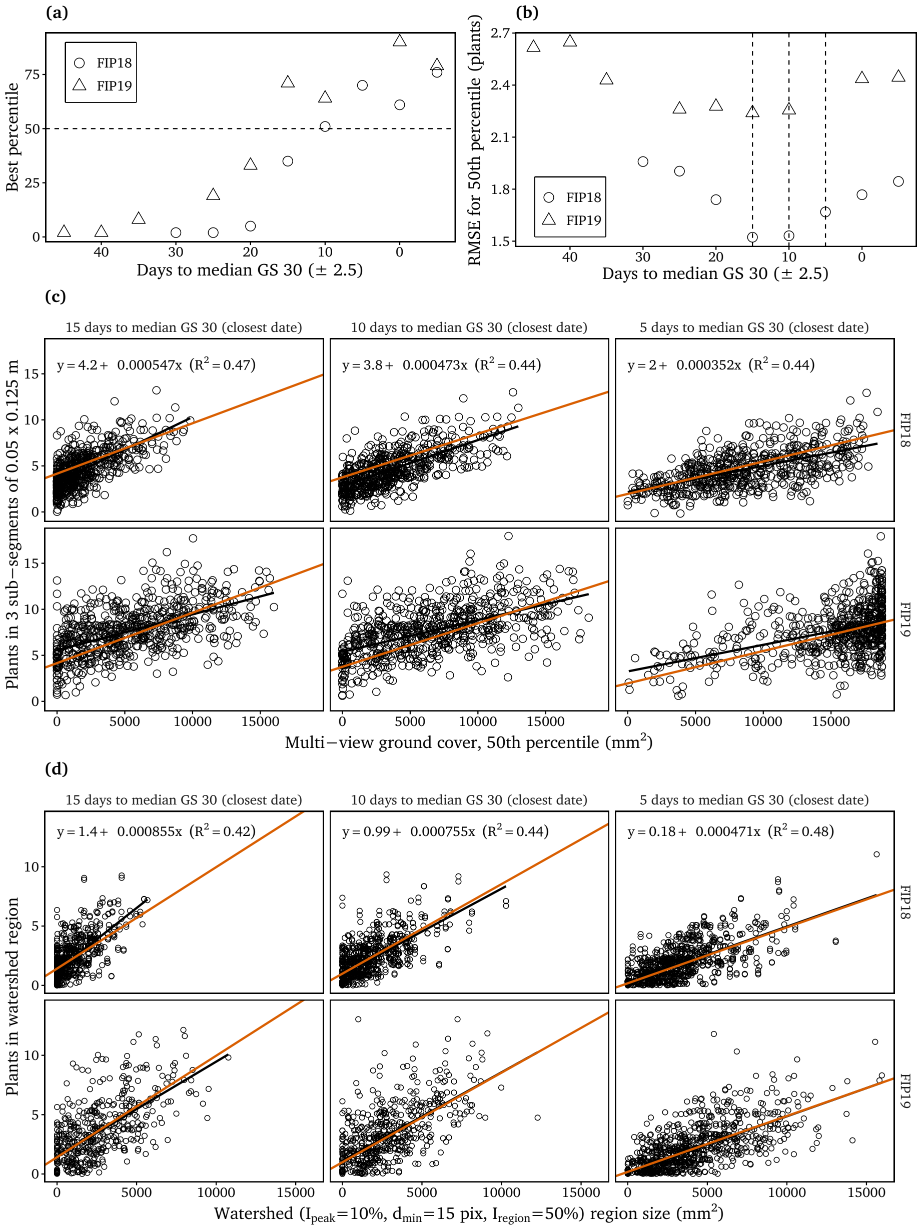

Figure 8: Threshold value and linear regression parameter determination for the plant count prediction method: Best ground cover percentile in dependency to GS 30 (a), root mean squared error (RMSE) for the 50th ground cover percentile as plant count predictor in dependency to GS 30 (b), relationship between the 50th ground cover percentile and plant counts for three time points (five, ten and 15 days before GS 30) (c), and relationship between watershed region sizes and plant counts for the same three time points (d). The dashed line in (a) represents the 50th ground cover percentile that was selected for (b), the dashed lines in (b) represent the three time points that were selected for (c, d). Solid black lines in (c, d) represent linear regressions for the specific time point and year-site, solid red lines the year-site-independent linear regressions.

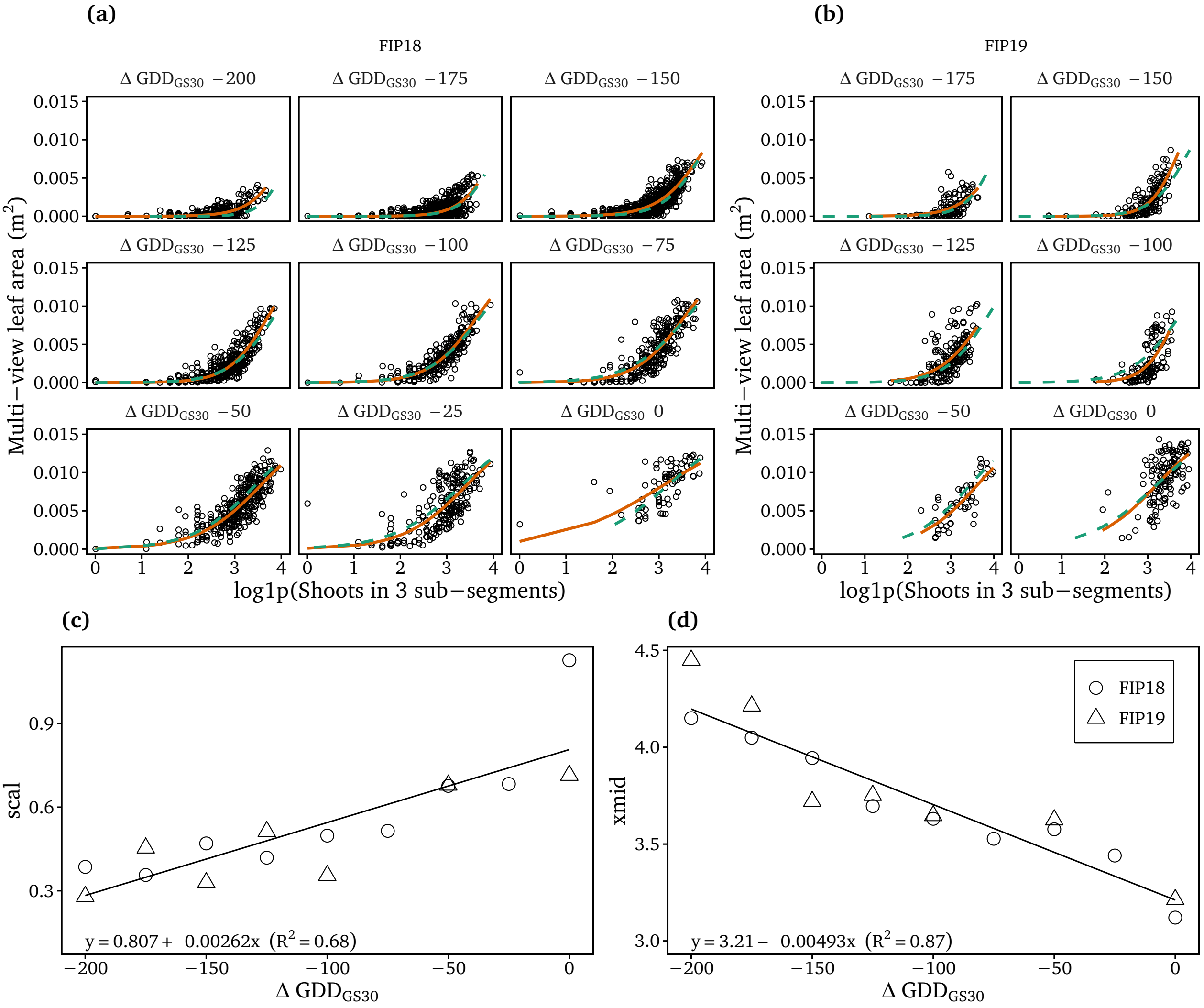

Figure 9: Empirical determination of logistic relation between multi-view based leaf area and shoots for the shoot count prediction method: Non-linear fit of logistic curve to leaf area data in dependency of the distance to GS 30 in GDD (ΔGDD_{GS30}) for two year-sites (a, b) and linear dependency of the estimated logistic parameters *scal* (c) and *xmid* (d) on ΔGDD_{GS30}. Solid green lines in (a, b) represent non-linear fits for the specific time point and year-site, dashed red lines the year-site-independent non-linear fits.

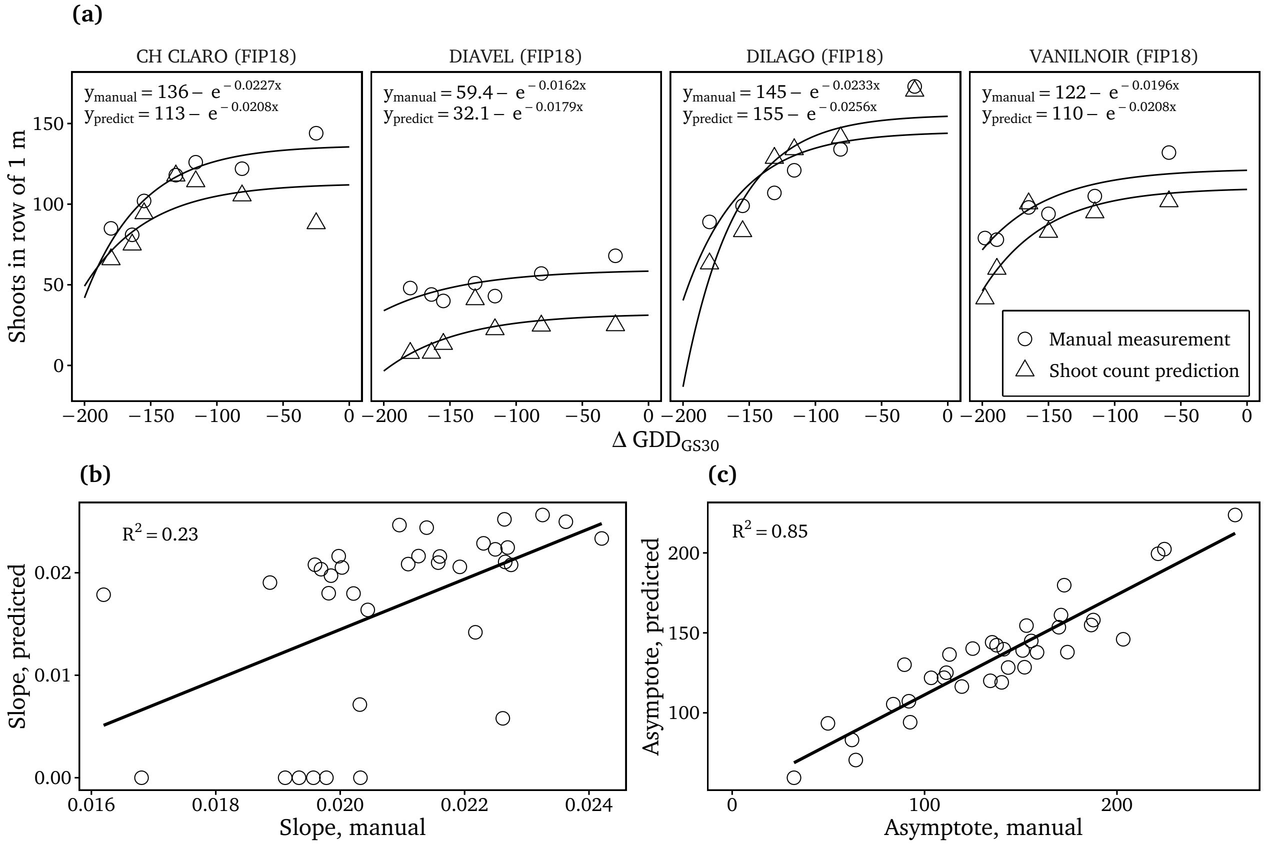

Figure 10: Shoot count dynamics: Non-linear model fit to manual reference measurements and predictions for four selected genotypes at year-site FIP18 (a), and fitted parameters tillering rate (Slope; b) and shoot count before cessation (Asymptote; c) for all genotypes at year-site FIP18.

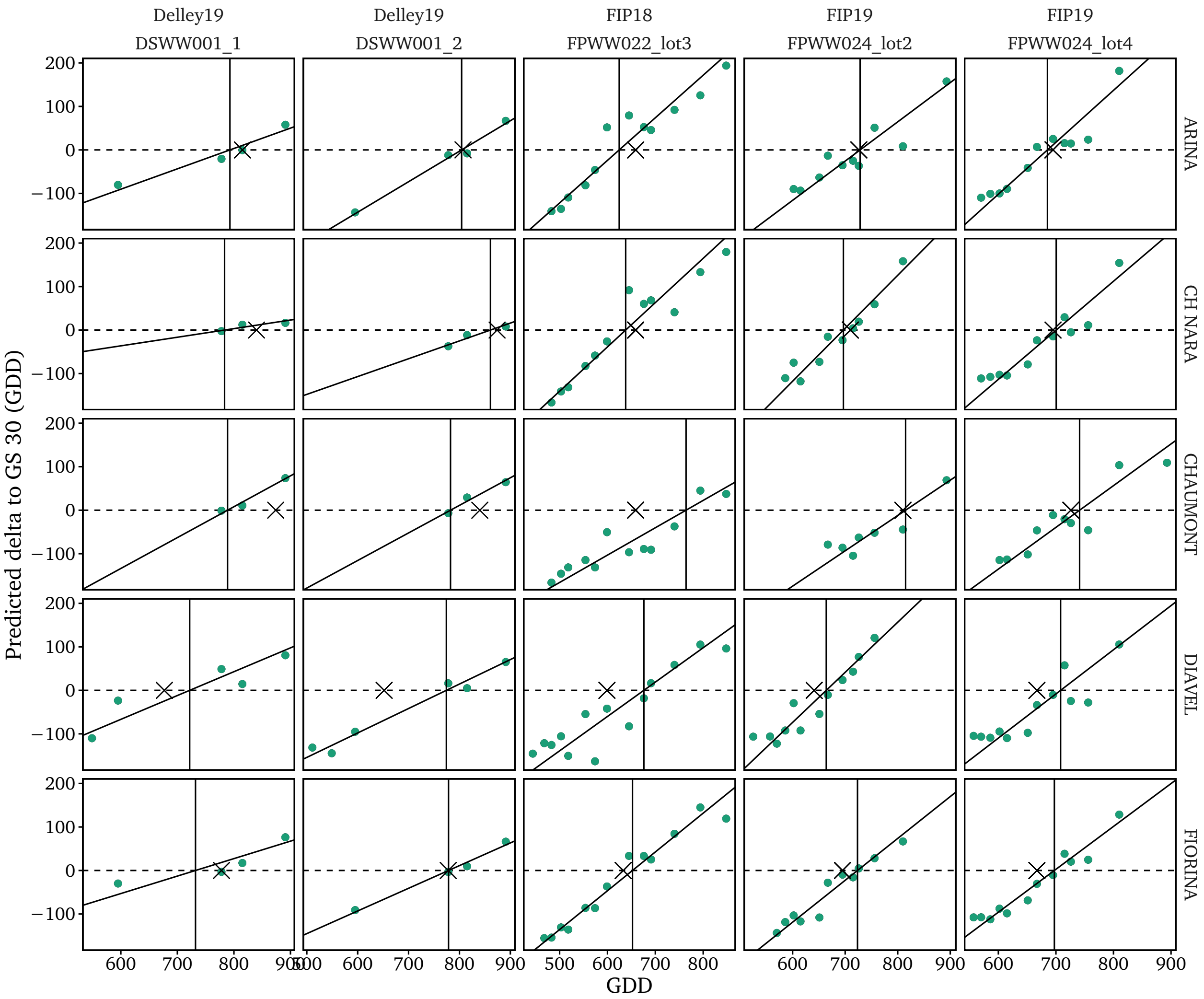

Figure 11: Beginning of stem elongation dynamics: Linear regression fit for five selected genotypes for all year-sites and one (FIP18) respectively two (FIP19, Delley19) replications. Green points represent predicted delta to GS 30 values, skewed solid black lines linear regressions, vertical solid black lines predicted GS 30 turning points based on regressions, and black crosses manual determined GS 30 turning points.

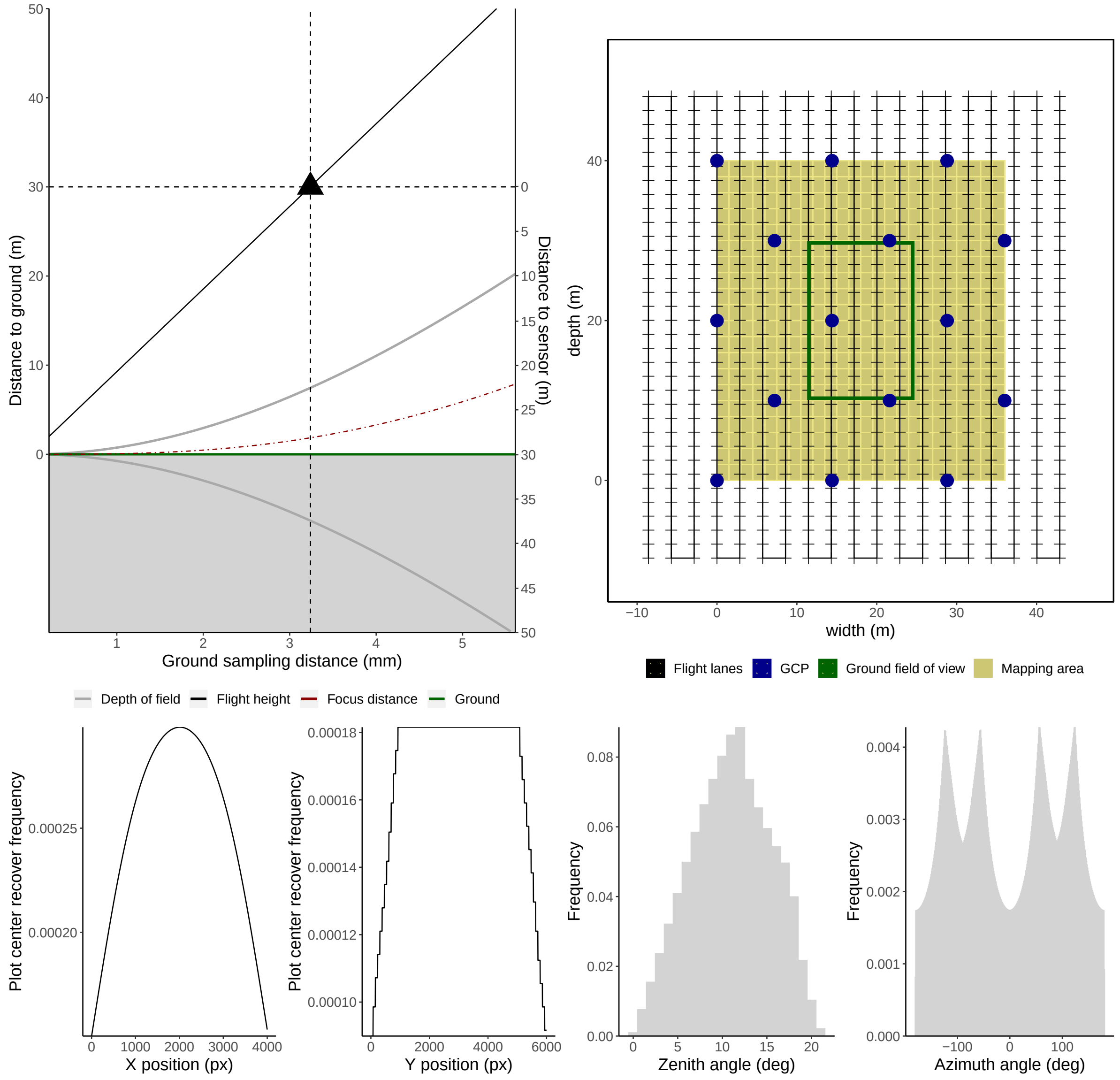

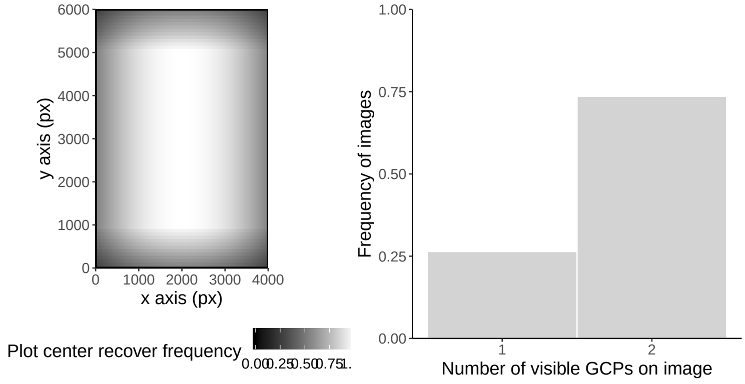

	Position (x)	Position (y)	Recover frequency
GCP 1	0	0	18
GCP 2	0	20	33
GCP 3	0	40	18
GCP 4	7.2	10	44
GCP 5	7.2	30	44
GCP 6	14.4	0	30
GCP 7	14.4	20	55
GCP 8	14.4	40	30
GCP 9	21.6	10	44
GCP 10	21.6	30	44
GCP 11	28.8	0	30
GCP 12	28.8	20	55
GCP 13	28.8	40	30
GCP 14	36	10	22
GCP 15	36	30	22

Figure 12: Phenofly Planning Tool report.
