# Supplementary material for: Repeated Multiview Imaging for Estimating Seedling Tiller Counts of Wheat Genotypes Using Drones
Source: Plant Phenomics. 2020 Sep 7;2020:3729715. doi: 10.34133/2020/3729715 (PMC7706335; doi:10.34133/2020/3729715)
Supplement: Supplementary Materials — A: additional tables. B: additional figures. C: site description. D: high-throughput processing details. E: plant count method details. [file 3729715.f1.zip › 3729715.f1/S_C_Site_description.pdf]

Supplementary Materials

C: Site description

Site FIP

The site FIP is located at the ETH research station of agricultural sciences in Lindau Eschikon, Switzerland (47.449 N, 8.682 E, 556 m a.s.l.). The soil type is an eutric cambisol consisting of 21% clay and 21% silt. Humus content is 3.5% and pH 6.7. Soil characteristic were determined in 2015 (Eric Schweizer AG, Thun, Switzerland). Preliminary to wheat (*Triticum aestivum L.*), soybeans (*Glycine max* (L.) Merr.) and buckwheat (*Fagopyrum esculentum* Moench) were grown. After preliminary crops were harvested, the soil was plowed and harrowed before wheat was drill-sown.

FIP18:
The design was an augmented row-column design with 351 test varieties (replicated twice) and 3 check varieties (replicated 18 times). The experimental unit was a plot of 1.6 m^2 (1.5 m in row direction; 2 m in range direction including paths and wheal tracks). The two replications of the test varieties were allocated to different lots of the FIP crop rotation. Each lot contained 18 columns (here called ranges) by 21 rows. The test varieties were allocated in a row-column design with incomplete blocks of 1 row by 18 ranges and 6 ranges by 42 rows (i.e. three range sections spanning across both lots). The first dimension covered the variability in working direction of the machinery (driving within ranges along rows). The second incomplete block dimension covered the position in the slightly sloped lots by distributing them into an upper, central and lower range section. The check varieties were allocated to each lot in 9 complete blocks of size 42 (6 ranges by 7 rows) each holding all three checks similarly. Each row and range was covered by 1 to 2 checks. The 36 test varieties were a subset of the overall test set of 351 genotypes. The design was generated using the R-package DiGGER.

Wheat was sown in 9 rows per plot with a row length of 1.7 m and a row distance of 0.125 m on October 17, 2017. Sowing density was 400 plants m^{-2}. One day after sowing, herbicide (Herold SC, Bayer AG, Leverkusen, Germany) was applied to ensure weed free plots. Several fungicides and insecticides were applied in spring to ensure healthy plants.

On February 28, 2018, April 17 and May 14, 52 kg nitrogen (N) ha^{-1}, 72 kg N ha^{-1} and 12 kg N ha^{-1} were applied. Additionally, in total 52 kg phosphorous ha^{-1}, 93 kg potassium ha^{-1} and 21 kg magnesium ha^{-1} were applied.

FIP19:
For the design, 36 test varieties were arranged in four replications equally allocated across two lots of the FIP. The experimental unit was a plot of 6.1 m^2 (1.5 m in row and 6 m in range direction including paths and wheal tracks). The varieties were allocated in a row-column design as follows: Full replicates in row direction consisted of six rows by six ranges. Blocks in range direction consisted in two ranges spanning the 24 rows of both lots, thus holding 1.33 replications per genotype. This dimension was introduced to cover the spatial trend of the sloped field in an upper, central and lower part. The design was generated using the R-package DiGGER.

Wheat was sown in 9 rows per plot with a row length of 5 m and a row distance of 0.125 m on October 17, 2018. Sowing density was 400 plants m^{-2}. One day after sowing, herbicide (Herold SC, Bayer AG, Leverkusen, Germany) was applied to ensure weed free plots. Several fungicides and insecticides were applied in spring to ensure healthy plants.

On February 27, 2019, April 8 and May 27, 52 kg N $^{-1}$, 72 kg N $^{-1}$ and 24 kg N ha^{-1} were applied. Additionally, in total 92 kg phosphorous ha^{-1}, 120 kg potassium ha^{-1} and 15 kg magnesium ha^{-1} were applied.

Site Delley (Genevey 2)

The site Delley was located at Delley, Switzerland (46.918 N, 6.979 E, 500 m a.s.l.). The soil had a clay content of 15-25%, humus content was 1.5% and pH 7.2. Soil characteristic were determined in 2017 (Sol Conseil, Nyon, Switzerland). Preliminary to wheat, pea (*Pisum sativum* L.) were grown. After preliminary crops were harvested, the soil was irrigated with 20 mm water, plowed and harrowed before wheat was drill-sown.

Delley19:
For the design, varieties were arranged in a 6 x 6 lattice design with four replications. The experimental unit was a plot of 6.1 m^2 (1.5 m in row and 6 m in range direction including paths and wheal tracks).

Wheat was drill-sown in 9 rows per plot with a row length of 5 m and a row distance of 0.125 m on October 24, 2018. Sowing density was 350 plants m^{-2}.

On February 25 and April 1, 84 kg N $^{-1}$ and 65 kg N $^{-1}$ ha^{-1} were applied. Additionally, in total 50 kg phosphorous ha^{-1}, 75 kg potassium ha^{-1}, 20 kg calcium ha^{-1} and 18 kg magnesium ha^{-1} were applied.
