# Supplementary material for: Repeated Multiview Imaging for Estimating Seedling Tiller Counts of Wheat Genotypes Using Drones
Source: Plant Phenomics. 2020 Sep 7;2020:3729715. doi: 10.34133/2020/3729715 (PMC7706335; doi:10.34133/2020/3729715)
Supplement: Supplementary Materials — A: additional tables. B: additional figures. C: site description. D: high-throughput processing details. E: plant count method details. [file 3729715.f1.zip › 3729715.f1/S_D_High_throughput_processing_details.pdf]

Supplementary Materials

D: High-throughput processing details

Image processing for 34 measurement campaigns at three different year-sites was done in a high-throughput manner without human interaction. Thereby, SfM processing on a 64-bit Windows 10 PC with two Intel Xeon 3.1 GHz CPUs, two NVIDIA GeForce GTX 970 GPUs and 256 GB RAM took 18 hours for the first campaign that included dense point and digital elevation model generation, and 15 minutes for each of the following campaigns. The segmentation of images of one campaign on a 64-bit Windows 7 PC with Intel Xeon 3.5 GHz CPU and 128 GB RAM took 10 hours, while the processing time used for the extraction of features from multi-view images was less than one hour for all campaigns. In summary, the processing from images to extracted plot-based values was feasible in less than half a day processing time per campaign with minimal overall user interaction of approximately ten minutes per campaign. Nevertheless, complete processing took approximately two weeks computational time and six hours human interaction in total.

Reported accuracies are thereby for image alignment between 1 to 2 pixel and for GCP alignment between 0.005 to 0.015 m in XY and 0.05 m in Z (Roth, 2018).

References

L. Roth. PhenoFly Sample Dataset 2: RGB Mapping Flight at 28 m. ETH Zürich, 2018. doi: 10.3929/ethz-b-000269882.
