# Supplementary material for: Repeated Multiview Imaging for Estimating Seedling Tiller Counts of Wheat Genotypes Using Drones
Source: Plant Phenomics. 2020 Sep 7;2020:3729715. doi: 10.34133/2020/3729715 (PMC7706335; doi:10.34133/2020/3729715)
Supplement: Supplementary Materials — A: additional tables. B: additional figures. C: site description. D: high-throughput processing details. E: plant count method details. [file 3729715.f1.zip › 3729715.f1/S_E_Plant_count_method_details.pdf]

Supplementary Materials

E: Plant count method details

Ground cover percentile as predictor

1. It was assumed that a certain multi-view ground cover percentile ($mvGC_i$) correlates well with plant counts (N_{plants}) (Figure 3b1-2).

To determine the most robust predictor, we examined the linear relationship between percentiles and manual sub-segment plant count measurements (Section 2.2.2) for a range of measurement days before GS 30, while two plots in 2018 where excluded due to high freezing damage (rating >4). To reduce errors introduced by small positioning shifts in manual counting, sub-segments were aggregated using a moving window with the size of three sub-segments. Then, the best percentile per group of ± 2.5 days to GS 30 was evaluated using standard deviations of linear fit residuals (Supplementary Materials B, Figure 8a).

Results revealed that low percentiles should be favored in early growth phases, but higher percentiles for days close to GS 30 (Supplementary Materials B, Figure 8a). Consequently, we selected the "neutral" 50th multi-view ground cover percentile as predictor. To evaluate more specifically which measurement dates are favorable when using the 50th percentile, we calculated root mean squared errors (RMSEs) for all measurement days (Supplementary Materials B, Figure 8b). RMSEs showed that data from five, ten and 15 days before GS 30 serve best as estimation base for plant counts. Nevertheless, if using the ground cover percentile approach a year effect was expected (Supplementary Materials B, Figure 8c) and confirmed by an ANOVA test (Supplementary Materials A, Table 3).

Local maxima as predictor

2. It was assumed that local maxima counts in multi-view images correlate with true plant counts (Figure 3b1,3).

Using a search grid, both parameters were optimized by minimizing the error between true plant counts based on sub-segment measurements and number of determined local maxima, which favored values of 10 % for I_{peak} and of 15 pixels for d_{min}.
